# Model of dimensions and variables of corporate social responsibility updated through structural equations

**DOI:** 10.1371/journal.pone.0296761

**Published:** 2024-06-25

**Authors:** Miguel A. Bustamante-Ubilla, Mauricio Carvache-Franco, Orly Carvache-Franco, Wilmer Carvache-Franco

**Affiliations:** 1 Facultad de Economía y Negocios, Universidad de Talca, Talca, Chile; 2 Sistema de Posgrado, Universidad Católica de Santiago de Guayaquil, Guayaquil, Ecuador; 3 Universidad Bolivariana del Ecuador, Durán, Ecuador; 4 Universidad Espíritu Santo, Samborondón, Ecuador; 5 Facultad de Ciencias Sociales y Humanísticas, Escuela Superior Politécnica del Litoral, ESPOL, Guayaquil, Ecuador; Wroclaw University of Science and Technology: Politechnika Wroclawska, POLAND

## Abstract

Corporate social responsibility (CSR) is a constantly evolving concept that reflects changes in society and the expectations of stakeholders in a process that leads companies to adapt to respond to new challenges and demands. Similarly, the theory and practice of CSR have moved from regulatory compliance to a more holistic approach that requires more sophisticated models that allow for a deeper understanding. Following the fact that companies now have access to a greater amount of information related to their social and environmental performance, as well as comparative data from other organizations, which is why it is necessary to have a management tool that allows collecting, process, and generating more accurate and meaningful metrics that more effectively reflect a company’s impact on society. Consequently, the present work aims to identify a system of variables and dimensions representative of the management of organizations that allows the validation of a generic model of corporate social responsibility determined through factorial and structural modeling capable of reflecting more accurate and up-to-date dimensions and metrics of model variables and dimensions. To carry out the study, a sample of 667 middle and senior managers from medium and large companies in Guayas, Ecuador, were randomly contacted in their respective business contexts by trained interviewers. The data processing was carried out in stages. First, the exploratory factorial analysis method of the items was applied to form relevant factors. Then, the dimensions’ structural modeling was formulated and ratified through the pertinent goodness-of-fit indices. The results determined a system of correlated factors whose items present estimators of commonality and high and significant factorial loads, excluding variables that did not exceed the sensitization criteria applied. Finally, a model made up of 15 factors and 66 variables ratified using the comparative adjustment indices, the chi-square likelihood ratio, and the mean square error of approximation is introduced, confirming the proposed corporate social responsibility model.

## 1. Introduction

In general, the literature shows a growing demand for information and transparency about the social and environmental performance of companies [1], as well as government agencies and other social institutions [2] that have progressively assumed various types of responsibilities [3,4] thereby assuming the need to identify which are those CSR variables that must or should be included in their respective management areas and that this work tries to answer.

What began as philanthropy has led to the development of activities with a social impact beyond profits [2] and legal requirements and obligations assumed with third parties [2], including, among others, the obligation to mitigate damages and maximize the benefits reported to society [3]. Hence, progressively, a taxonomy of concepts has been formed that categorizes CSR theories [1] as diverse and complementary. First, postulating concepts related to the economic, social, environmental and ethical, giving rise to a new option of self-regulated management of organizational activities [1].

Secondly, in recent times the appearance in the literature of conceptual and instrumental models [2] that seek to identify and measure factors and variables related to the social, political and environmental dimensions that are relevant for companies according to their respective business orientations [3], for example, when seeking to balance cause and effect relationships between economic, social and environmental dimensions. Thirdly, although tangentially, political theory underscores the social power of the groups that influence the socio-economic spheres and society as a whole, demanding greater data availability from companies, as well as a growing demand for information. and transparency [3]. Finally, fourthly, the theoretical and practical recognition acquired by the ethical and moral foundations of business facts, which drive the need to investigate the existence of an updated management model that allows the identification of a set of significant variables and a more accurate measurement. accurate and relevant performance of companies, which in turn promotes the continuous improvement of the dimensions of corporate social responsibility that this work seeks to propose [3].

Consequently, the managers of the organizations need to have some instrument that allows them to collect these perceptions [1] and that the present work seeks to make available to the organizations [2].

From a theoretical perspective and considering that society integrates people, organizations and other actors that compose it, it is necessary to quote Rawls [4], since it is people, in their individuality, who express their needs and potentialities [5] creating organizations together with other individuals [6] and promote the emergence of companies [2] to serve society as a whole [7,8]. Thus, the results of the organizations must derive from the effort and not from uncontrollable circumstances of an unpredictable general scenario [9].

In this context, it is necessary to identify which are the variables that organizations must manage [7] and that this work aims to determine from the perspective of corporate social responsibility [3,4,10], on the one hand, to promote acting and making humans [1,4,6] and, on the other, to that people and organizations can achieve their objectives in equal opportunities [7] and through the exercise of their respective talents and strengths [8,10]. However, there are those who promote equality of results [5–7] and who recognize the diversity of qualifications and efforts that consequently lead to different results [2].

Hence, the social responsibility of organizations incorporates among its decisions [2], various variables and dimensions [7], some relevant for society as a whole, such as environmental ones, and others that affect those who are part of the organizations [3], for example, through contracts [11], through coexistence agreements [8] that are expressed in each person uniquely [9]. On the one hand, there are those who seek to develop their activities in total freedom [8] and, others, who voluntarily link up with others [11,12], giving rise to new spaces for collaboration [13] such as those promoted by business organizations [14]. Consequently, and on this conceptual basis [2,10], organizations will seek to comply with their obligations [12], forcing themselves to contribute to the global quality of life [1,2,10] and that is precisely what the present study seeks. determine statistically.

In accordance with the above, it is possible to define a CSR management oriented to society as a whole [4,6,13]. In particular, collecting a set of variables and dimensions [2,10] capable of forming a system of key factors that respond to the various interest groups with which organizations relate [3,11,14]. This perspective is clearly supported by the theory of interest groups [3,11] according to which, organizations must deploy their capacities to meet their obligations [14,15], respond to demands and claims [3,8] and satisfy the counterparties in accordance with the financial, legal and contractual obligations that correspond to them [10,15].

## 2. Methodology

This section describes the sampling and details the statistical analyses steps of factorial and structural modelling. This study is part of the Project ethically approved by the Polytechnic University ESPOL of Ecuador. Informed consent was requested in writing at the beginning of the questionnaire.

### 2.1. Population and sample

The population was made up of professionals, including middle and high managers of medium and large companies in Guayas, Ecuador. The researchers considered the minimum number of contacts per item (n/p = 10:1; n/p = 5:1) to determine a minimum reach contact [16]. First, prior analysis procedures were applied to calculate an a priori sample through the G*Power 3.1 program, considering a finite population to define the power parameters and effect size for a sample group [17]. Consequently, an inclination of 0.015, a probabilistic error of 0.05, and a reliability of 0.95 was determined for an a priori sample of 472 cases.

### 2.2. Process

The contacts were randomly selected as normal respondents from the business contexts of Guayas, Ecuador, forming a relevant sample [18] accessed through some virtual channels. In the piloting stage, the questionnaire was applied to 35 managers, ensuring they were not included in the final study sample [19]. The team of researchers carried out the definitive field work through virtual means, which made it possible to contact an adequate number of interviewees, considering an average response time of 25 to 35 minutes. The data collection was carried out between November and December 2021 under the anonymity and confidentiality regulations established by law, with the prior informed consent of the interviewees.

### 2.3. Research instrument

The original instrument to collect the perceptions of importance of middle managers and senior managers on a system of variables and dimensions of CSR, was developed in the context of a doctoral thesis [20] of one of the co-authors, which by the way was duly validated, precisely because of its ability to measure the variables that were identified in the 1990s to measure CSR in the Chilean context [12]. The instrument made it possible to give shape and content to a construct made up of representative factors of the social concerns of managers about business organizations and which were expressed in terms of their relative importance [10,12]. This is how a structured questionnaire with statements and answers on a 5-point Likert-type unidirectional scale was shaped. The CSR model implicit in the instrument took into account the business and social reality in addition to the changes that were perceived in the Chilean business context at the time. However, a very different reality is currently observed, which highlights the need to update the instrument, proving its effectiveness in another context, this time in the Ecuadorian reality [21] and in which the business organizations that are part of the sample determined for this study are inserted.

The dimensions determined by the original instrument [20] were the following: F1, Economic and financial situation; F2, Commercial situation; F3, Production; F4, Productivity; F 5, Personnel; F6, Customer Service; F7, Community relations; F8, Security; F9, Remuneration and incentives; F 10, Job satisfaction;, F11, Participation and communications; F12, Training and development; F13, Employment benefits; F14, Working conditions; F15, Business management; F16, Relations with the national and international community and, F17, Environment and ecology. These 17 factors structure a CSR construct that provides senior and middle managers with a set of 104 variables that they must evaluate using the 5-point Likert scale [20]. This is how the 104 variables grouped into 17 factors were presented to the interviewees to collect their perceptions, precisely to identify the CSR components that are currently validated as relevant and allow to consolidate and, eventually, focus the original instrument [20] in a number of variables capable of forming a system of items and representative factors of CSR updated to the reality of medium and large organizations and in the Ecuadorian context [21].

### 2.4. Study hypothesis

As has been developed in the previous sections, CSR has been studied theoretically in various contexts and perspectives [2,3,12] as well as through quantitative methods with cross-sectional and correlational descriptive designs [22,23] managing to make available from the academic community [20,24], various statistically validated constructs that identify items, as well as factorial groupings that guide the best management of CSR by organizations [3,4]. Certainly these contributions were made in relation to a certain time and place, however, at present it is observed that society has evolved towards new and diverse relevant issues such as those derived from communications [13], the relevance of human capital [15], the incorporation of diversity and women into corporate governance [25–27] and the prominence of environmental management [21,24] among others. That is why the team of researchers presenting this work uses as a basis for analysis the instrument previously developed by one of its co-authors [20] and assumes the objective of identifying a system of variables and updated dimensions of CSR that allows proposing a construct duly validated, on this occasion, in the Ecuadorian context. Consequently, and based on the theory and practice of CSR presented, the following two hypotheses are proposed:

H1: The CSR variables identified in this work make up representative factors of corporate social responsibility, explaining more than 50% of the total variance.
H2: The constitutive factors of CSR determined in this study are coherently related and covary among themselves, forming a robust system of dimensions duly ratified by means of statistically significant goodness-of-fit indices.

### 2.5. Factorial and structural model of the CSR variables

In order to identify the representative variables and dimensions of the management of organizations capable of forming a generic CSR model [2,10,12], an exploratory factor analysis was first carried out, AFE of the items in order to generate a system of representative factors of the reality under study [17–19]. Secondly, and from the procedural perspective, the Pearson dispersion matrix and the varimax rotational extraction method [16,17] were used for factor determination. Likewise, since the responses are collected on a 5-point Likert scale [28], it can be assumed that the responses can behave in an approximately normal manner [18]. Likewise, and in accordance with the objective of identifying a system of robust variables and factors, we proceeded to verify the key inclusion parameters of the variables to their respective factors. For this, the psychometric analysis of the items was carried out, which were evaluated through the communality indices (≥ 0.40) and factorial load (≥ 0.50) of the variables to their respective factors. In turn and to confirm the reliability of the instrument, the Cronbach’s Alpha index was verified, requiring a high and consistent estimator (≥ 0.80) [29].

Regarding the objective of validating the previously determined factors, the factors were processed using Exploratory Structural Modeling of Structural Equations (ESEM) [18]. For this, the items included in the factors [20] were analyzed according to the steps suggested in the confirmatory methods [30]. In this context, the researchers sensitized the estimators of the variables and the significance indices (***), to confirm that the items are part of the determined factors [31] and represent with some certainty the reality analyzed [18,31]. Finally, the findings were ratified through the respective goodness-of-fit indices [29,31] after verifying that the estimators meet the required requirements. Notwithstanding the above, it is necessary to indicate that the ESEM analysis delivers statistically significant results when it is unlikely that these are due to chance [19,29]. To do this, the goodness of fit indices were reviewed [32,33]. The comparative fit index (CFI) was verified, which must be located in the acceptance range (≤ 0.90) [34], and the Chi-Square Absolute Statistical Likelihood Ratio Index (CMIN/DF) was verified, which must be reached. the necessary level (≤ 3) to end with the Mean Square Approximation Error Index (RMSEA) whose estimator must be less than 5% (≤0.05) [16,30].

### 2.6. Use of software

Based on the fact that the present study aims to identify variables and dimensions capable of forming a generic CSR model, it was decided to obtain the sample for the study in two phases. In the first place, the determination of an a priori sample was obtained by using the G*Power 3.1 program, which has the capacity to ensure the minimum parameters of statistical estimation so that it is representative [18]. Subsequently, once the data collection instrument was applied, the number of cases collected was verified, which must be equal to or greater than the previous sample and through which the estimators of the sample called expost [19] were confirmed.

Next, the data processing for the factorial analysis, the statistical software SPSS V23 was used. Next, to carry out the ESEM structural modeling, the covariance method was used, which was carried out using the SPSS AMOS program [16,17,35].

## 3. Results

The description of the research phases begins by detailing the sample of executives and managers interviewed followed by the factorial modeling of the dimensions and variables of corporate social responsibility, in addition to the description of each of the determined factors. Then, the structural modeling of dimensions and variables is presented and, finally, the measurement model of the factors that confirm the CSR factors and variables that this paper proposes.

### 3.1. Sample description

The sample determined for the study reached 667 cases, among which 55.5% are men. The distribution by age shows that 82.7% are over 30 years of age. While the majority level of education was professionals with 51.8% followed by university students who reached 48.2%. The marital status of married reached 55.5% and that of single 37.7%. While the positions of the interviewees were distributed between managers who reached 51.4% and managers who reached 48.6%. The years of service of those who indicate less than 10 years is 20.5% and of those who register more than 10 years it is 79.5%. On the other hand, the type of permanent contract concentrates a majority of 58.2% and, finally, the size of companies is distributed between the large ones that are 46.7% and the medium ones that reach 53.3% (Table 1).

**Table 1:** Description of the sample.

Sex		Age		Education level		Maritalk status	
N	667		667		667		667
Man	55.0	≤ 30	17.3	Professionals	51.8	Separated	6.8
Women	45.0	≥ 30	82.7	College	48.2	Single	37.7
						Married	55.5
Post		Years of service		Type of contrat		Compeny size	
	667		667		667		667
Middle manager	51.4	≤ 10	20.5	For hire	41.8	Big	46.7
Senior manager	48.6	≥ 10	79.5	Indefinite	58.2	Median	53.3

### 3.2. Factorial modelling of dimensions and variables of corporate social responsibility

As a first step, the exploratory factorial analysis of the data was carried out applying the method of principal components, with Varimax rotation and Kaiser normalization, reaching convergence after 25 iterations. As detailed in Table 2, the adequacy measure obtained through the Kaiser-Meyer-Olkin (KMO) index reached 0.787, followed by a Bartlett sphericity test that gave a Chi-square of 18023.258 with 5356 degrees of freedom and a significance index at 1% (0.000). The instrument’s reliability using Cronbach’s alpha was 0.892, considered high.

**Table 2:** Factorial model of corporate social responsibility Ecuadorian sample of 667 cases.

**F1 Environment and ecology**			Factor	**F2 Work satisfaction**			Factor
Code and Variable	Explained Variance	**11.927**	Loading	Code and Variable	Explained Variance	**9.508**	Loading
G16V9	Foreign trade		0.730	G10V2	Psychological balance		0.830
G17V7	Energy saving		0.700	G10V3	Work Absenteeism		0.818
G17V6	Threats to Population Health		0.674	G11V1	Labor Relations		0.814
G17V2	Acidity Or Alkalinity of The Waters		0.664	G10V4	Staff Rotation		0.692
G17V5	Pollution		0.648	G11V3	Participation System		0.646
G16V4	Advertising’s Social Responsibility		0.612	G11V2	Freedom of opinion		0.637
G17V1	Environmental Conservation		0.610				
G17V4	Residues and Waste		0.562				
G16V10	International Competitiveness		0.528				
**F3 Corporate management**			Factor	**F4 Production**			Factor
Code and Variable	Explained Variance	**5.400**	Loading	Code and Variable	Explained Variance	**4.014**	Loading
G14V6	Financing of Social Works		0.649	G3V2	Quantity		0.647
G15V5	Research and development		0.772	G3V3	Stocks		0.622
G15V6	Direct and Indirect Employment Generated		0.723	G3V4	Costs		0.680
G16V7	Community Welfare		0.717	G4V1	Technical Productivity		0.747
G16V2	Social Responsibility Information		0.599	G4V3	Productivity Factor		0.593
				G5V1	Template		0.674
**F5 Working conditions**			Factor	**F6 Compensation and incentives**			Factor
Code and Variable	Explained Variance	**3.409**	Loading	Code and Variable	Explained Variance	**3.045**	Loading
G14V2	Shift Assignment		0.718	G5V3	Training, Promotion and Security		0.614
G14V3	Technology		0.682	G9V3	Ascents and Promotions System		0.744
G14V4	Labor day duration		0.726	G9V5	Staff Parties		0.635
G14V5	Resources Guarantee for Older Workers		0.764	G9V6	Cultural aspects		0.630
G15V2	Performance evaluation		0.634				
**F7 Participation and development**			Factor	**F8 Training by competencies**			Factor
Code and Variable	Explained Variance	**2.722**	Loading	Code and Variable	Explained Variance	**2.461**	Loading
G11V7	Complaints Treatment		0.729	G12V4	Holidays for Training		0.846
G11V8	Business Committees		0.789	G12V5	Learning Programs		0.884
G12V1	Training and Development		0.765	G12V6	Staff Advancement Pathways		0.791
G12V2	Freedom of opinion		0.703				
**F9 People Management**			Factor	**F10 Environment management**			Factor
Code and Variable	Explained Variance	**2.395**	Loading	Code and Variable	Explained Variance	**2.050**	Loading
G5V4	Wages		0.766	G7V1	Relationship with the Public Administration		0.771
G6V1	Clients Judgment		0.699	G7V2	Company image		0.720
G9V2	Merit Recognition		0.720	G9V11	Stock Purchase Option		0.615
**F11 Business management**			Factor	**F12 Employment benefits**			Factor
Code and Variable	Explained Variance	**2.012**	Loading	Code and Variable	Explained Variance	**1.933**	Loading
G15V7	Employment of Human and Material Resources		0.657	G13V11	Quotes		0.757
G15V8	Company Profitability		0.631	G13V12	Compensation Maternity Leave		0.789
G15V9	Company Expansion		0.585	G14V1	Working conditions		0.808
G15V10	Responsibility Centers		0.558				
**F13 Supplies and acquisitions**			Factor	**F14 Economic and financial situation**			Factor
Code and Variable	Explained Variance	**1.658**	Loading	Code and Variable	Explained Variance	**1.636**	Loading
G2V3	Link with the Environment		0.781	G1V2	Rotation Rate		0.654
G2V4	Purchasing Management		0.800	G1V3	Cost effectiveness		0.787
G3V1	Quality of supplies		0.807	G2V1	Sales		0.647
**F15 Family benefits**			Factor				
Code and Variable	Explained Variance	**1.553**	Loading				
G13V5	Home Loan System		0.752				
G13V6	Emergency Loans		0.641				
G13V7	Birth Assignments, Marriage, Deaths		0.509				
G13V8	Permissions and licences		0.579				
G13V9	Retirements		0.517				
**Total Explained Variance**			**55.723**	**Alfa Cronbach**			**0.892**

**Deleted variables**					
G1V1	Economic structure	G9V4	Advance System	G13V3	Special Bonus System
G1V4	Performance	G9V7	Sport activities	G13V4	Institutional Loans
G2V2	Distribution	G9V8	Punctuality in Payments	G13V10	Personnel Transportation
G4V2	Economic Productivity	G9V9	Holidays	G15V1	Organization Quality
G5V2	Social Climate	G9V10	Profit Sharing	G15V3	Corporate Responsibility Compliance
G6V2	Product quality	G9V12	Partial Unemployment Compensation	G15V4	Management Prestige
G6V3	Relationship with customers	G10V1	Work satisfaction	G16V1	Contribution To Government Regulations
G7V3	Participation In Community Activities	G11V4	Internal Information Magazine	G16V3	Company Prestige
G8V1	Job Security	G11V5	Communications	G16V5	Paid taxes
G8V2	Occupational Accident Prevention	G11V6	Union Life	G16V6	Financing of Social Works
G8V4	Temporary job	G12V3	Task Enrichment	G16V8	Consumer Satisfaction
G8V3	Occupational Diseases	G13V1	Health Fund	G17V3	Total Water Residues
G9V1	Remuneration System	G13V2	Compensation Fund		

For the determination of the factors of the CSR model, criteria of selectivity and persistence of the variables under study were included, measured through two criteria. First, the existence of a high index of communality (≥ 0.40) and, second, the determination of a factor load that is also high (≥ 0.50). Thus, it was possible to determine 15 factors that brought together a total of 66 variables of the 104 items analyzed from the original model. The resulting model explains 55.723% of the total variance, and the factors explain a significant proportion ranging between 11.927% for factor 1 and 1.553% for factor 15. Consequently, applying the respective estimators of each of the mentioned criteria of commonality and factorial load, 20 variables did not reach the necessary estimators of commonality, while 18 items did not achieve the necessary factorial loads, totaling 38 variables that did not meet the requirements to be part of the CSR model that this work seeks. Determine (Table 1).

Based on the variables that give shape and content to the 15 factors, a brief description of each of the factorial elements [17] of CSR that this model proposes and that was determined in the context of senior and middle managers was made. of medium and large companies in Guayas, Ecuador [21]. Furthermore, factor 1, environment and ecology, contains nine items (V1-V9) and explains 11.927% of the variance. It addresses the topics related to seven variables of the environment and pollution, most appreciated by the interviewees. These topics have a broad social impact, in addition to the demands of the international environment and competitiveness, precisely because they regulate companies’ activities in the origin market. Factor 2, job satisfaction, includes relevant items of internal management in organizations (V10—V15) that address key people management topics such as absenteeism, labor relations and participation, and it explains 9.508% of the variance. Next, corporate management, factor 3 highlights its importance through five variables (V16-V20) related to the financing of social projects, research and development, and social responsibility of information, which allows explaining 5.4% of the variance.

In this regard, from the perspective of internal management, factor 4, production, reveals the operational components of business activity through six items (V21-V26). These items deal with the quantity produced, stock, and productivity that explain key elements of the operations and that explain 4.014% of the total accumulated variance. Factor 5, working conditions, complements the model with five items (V27-V31) related to the hygienic factors and management of available resources that have a broad human impact. Such is the case of the item shifts, technology, and staff performance evaluation, which all explain 3.409% of the total variance. In addition, factor 6, compensation and incentives, training, promotion, and safety, as well as promotions variables (V32-V35), affect the behavior of personnel and explain a 3.045% variance.

Performing the analysis towards the intangible components of management, factor 7, participation and development, explains 2.722% of the total variance, through the administration of four essential variables (V36-V39) such as complaints handling, right to an opinion, and works councils. Similarly, factor 8, training by competencies, through three items (V40-V42) deals with key components such as learning programs and personnel progress paths, explaining 2.461% of the variance. Moreover, factor 9, people management, explains 2.395% of the total variance and presents one of the natural function’s organizations must manage. Factor 9 is executed through four variables (V43-V45) that allow managers to support the transformation processes and production, such as salary items, customer judgment and merit recognition.

Concerning external social responsibility, factor 10, environmental management, which explains 2.050% of the variance, is made up of three variables (V46-V48) that refer to the relationship between companies and public administration, the social image they achieve in society, and the stock purchase options that companies make available to their staff. Likewise, factor 11, business management, reveals four variables (V49-V52): employment, profitability, and expansion methods. These variables are implemented through specific responsibility canters typical of business activities, explaining 2.012% of the total variance. In addition, companies manage factor 12, labor benefits, which are essential for personnel at their various levels and hierarchies. It includes three variables (V53-V55) such as payment of contributions, compensation, and work conditions responsible for 1.933% of the variance. Finally, from the analysis of the last three factors, it can be seen that factor 13, supply and acquisitions, through three items (V56-V58), deals precisely with purchase management, acquisitions quality and their link with the environment, explaining 1.658% of the variance. Factor 14, economic-financial situation, brings essential variables companies’ survival (V59-V61), such as profitability, sales and inventory and capital turnover rates, which together explain 1.636% of the variance. Finally, factor 15, family benefits, through 5 variables (V62-V66), deals with aspects such as support systems for workers’ families through items related to emergency loans, housing, and various allocations. These items collaborate with the personnel’s life quality and are responsible for 1.553% of the variance. Synthesizing the analysis and as a result of the exploratory factorial modelling, 38 items out of the original 104 variables were excluded after applying the inclusion and exclusion criteria derived from the items’ commonality indices and the factor loadings of the variables [20]. The eliminated variables are detailed in Table 1, where the items’ groups and variable numbers are indicated. Although these variables registered adequate commonalities and factor loads (≥ 0.50), they did not form factors with at least three components to meet the moderate criterion of factor formation [32,33,36]. Consequently, these factors and their respective items were excluded from the study.

### 3.3. Structural modelling of variables and factors of corporate social responsibility

After verifying the factorial structure [17] of CSR in the Ecuadorian context that was described in the previous section, a second confirmatory phase of the model obtained was started, which was carried out through structural analysis [19]. In this stage of factorial ratification, the relationship analysis of the variables with their respective factors was carried out by means of the covariance method [31] whose results are presented in detail in Table 3.

**Table 3:** Model for measuring CSR variables and factors Ecuadorian sample of 667 cases.

**Variable**	**Estimators**	**S.E.**	**Standard**	**P**	**Variable**	**Estimators**	**S.E.**	**Standard**	**P**
G16V9	3.682	0.044	0.422	***	G10V2	4.013	0.041	0.597	***
G17V7	3.732	0.040	0.490	***	G10V3	4.319	0.030	0.731	***
G17V6	4.030	0.041	0.121	***	G11V1	4.028	0.037	0.495	***
G17V2	3.967	0.042	0.063	***	G10V4	4.196	0.032	0.638	***
G17V5	3.732	0.045	0.400	***	G11V3	4.394	0.031	0.437	***
G16V4	3.820	0.045	0.228	***	G11V2	3.954	0.038	0.473	***
G17V1	3.693	0.047	0.365	***	G14V6	3.387	0.049	0.492	***
G17V4	3.507	0.045	0.392	***	G15V5	3.204	0.057	0.502	***
G16V10	3.516	0.042	0.463	***	G15V6	3.501	0.048	0.498	***
					G16V7	3.448	0.046	0.111	***
					G16V2	3.319	0.058	0.340	***
G3V2	3.871	0.037	0.373	***	G5V3	3.577	0.048	0.319	***
G3V3	4.001	0.036	0.409	***	G9V3	4.064	0.039	0.474	***
G3V4	3.931	0.037	0.388	***	G9V5	4.240	0.035	0.594	***
G4V1	3.996	0.042	0.293	***	G9V6	3.915	0.042	0.415	***
G4V3	4.040	0.034	0.460	***	G11V7	3.144	0.045	0.417	***
G5V1	3.900	0.039	0.338	***	G11V8	3.100	0.043	0.468	***
G14V2	3.748	0.040	0.468	***	G12V1	3.447	0.041	0.783	***
G14V3	3.853	0.037	0.547	***	G12V2	3.766	0.041	0.496	***
G14V4	3.652	0.036	0.573	***	G12V4	3.402	0.044	0.792	***
G14V5	3.915	0.037	0.540	***	G12V5	3.367	0.042	0.862	***
G15V2	3.937	0.041	0.451	***	G12V6	3.415	0.042	0.571	***
G5V4	2.811	0.053	0.571	***	G13V11	4.327	0.037	0.434	***
G6V1	3.088	0.051	0.615	***	G13V12	4.508	0.031	0.584	***
G9V2	3.684	0.059	0.457	***	G14V1	4.520	0.030	0.636	***
G7V1	4.090	0.036	0.369	***	G2V3	3.975	0.034	0.463	***
G7V2	3.997	0.034	0.398	***	G2V4	4.321	0.031	0.563	***
G9V11	4.124	0.034	0.415	***	G3V1	4.394	0.031	0.547	***
G15V7	4.403	0.030	0.641	***	G1V2	4.675	0.025	0.504	***
G15V8	3.960	0.038	0.403	***	G1V3	4.115	0.036	0.246	***
G15V9	3.976	0.039	0.379	***	G2V1	4.493	0.029	0.384	***
G15V10	4.033	0.037	0.409	***					
G13V5	3.810	0.050	0.229	***	G13V8	3.940	0.040	0.350	***
G13V6	4.399	0.035	0.472	***	G13V9	4.420	0.031	0.592	***
G13V7	4.223	0.036	0.433	***					
Standardized estimators of CSR factors									
Factor	Estimators	S.E.	C.R.	P	Factor	Estimators	S.E.	C.R.	P
F1	0.533	0.034	15.877	***	F9	0.955	0.076	13.923	***
F2	0.448	0.028	16.236	***	F10	0.311	0.029	10.780	***
F3	0.774	0.052	14.909	***	F11	0.379	0.026	14.638	***
F4	0.347	0.025	13.947	***	F12	0.374	0.026	14.212	***
F5	0.506	0.031	16.562	***	F13	0.358	0.026	13.956	***
F6	0.491	0.033	14.724	***	F14	0.210	0.018	11.666	***
F7	0.563	0.047	11.907	***	F15	0.383	0.026	14.686	***
F8	0.916	0.061	16.692	***					

Nota: S.E. = Standard deviation; P = Stadistical significance (***); C.R. = Critical ratios.

The measurement model of variables and factors in Table 2 describes a system of estimators that reach relevant significance levels at 1% (***) and at the same time, the critical ratios are statistically significant since they reach C.R. greater than 1.96 [18,19]. Thus, for example, the results show that the 66 variables of the corporate social responsibility model reach estimators located in their range (0–1) with significant indices (***). Consequently, as shown in the lower part of Table 3, the 15 determined factors also present standard estimators located in their range (0–1) with their respective significances (***). Consequently, the structural modeling estimators confirm a system of duly related CSR factors that allow for a positive response to H1 insofar as they allow explaining more than the total variance (≥ 0.50) and therefore ratify the factorial analysis carried out [29,31].

The factorial model obtained in the first phase of the analysis (21) was confirmed from the determination of the estimators of the critical ratios C.R. and the significance indices P (***) determined by the structural analysis [31], whose estimators obtained, for each of the 66 variables incorporated into the factors of the CSR model, allow us to assume that the factor system behaves in a coherent manner with the statistical criteria of structural analysis [32].

### 3.4. Weighted model of CSR factors

To determine the percentage weights taken to 100% of the factorial system that make up the 15 factors that give shape and content to the CSR model, the level of intensity of each of the factors was verified according to the distribution of the variables and they were determined. the variance relationships (<---) of each of the 66 variables with each of the CSR factors determined CSR [29,31]. Thus, through the analysis of variance of the variables with their respective factors, it was possible to determine a system of estimators that are detailed in Table 4. As can be seen, the standardized estimators [32,34] present high values and located in its range (0–1), reaching indices that are between 0.479 as the minimum value (V62 <-- -F15) and 0.928 as the highest value (V41 <---F8), which confirms, once again, the existence of a relationship and the content of the factors [19,30] and the consistency of the CSR model achieved.

**Table 4:** CSR variables measurement model on each factor Ecuadorian sample of 667 cases.

**Variance ratios**	**Standard**	**Variance ratios**	**Standard**	**Variance ratios**	**Standard**
V1 <--- F1	0.650	V10 <--- F2	0.626	V16 <--- F3	0.702
V2 <--- F1	0.700	V11 <--- F2	0.855	V17 <--- F3	0.600
V3 <--- F1	0.694	V12 <--- F2	0.703	V18 <--- F3	0.706
V4 <--- F1	0.682	V13 <--- F2	0.799	V19 <--- F3	0.746
V5 <--- F1	0.633	V14 <--- F2	0.848	V20 <--- F3	0.583
V6 <--- F1	0.626	V15 <--- F2	0.688		
V7 <--- F1	0.604	V27 <--- F5	0.684	V32 <--- F6	0.565
V8 <--- F1	0.626	V28 <--- F5	0.740	V33 <--- F6	0.688
V9 <--- F1	0.681	V29 <--- F5	0.757	V34 <--- F6	0.771
V21 <--- F4	0.611	V30 <--- F5	0.735	V35 <--- F6	0.644
V22 <--- F4	0.640	V31 <--- F5	0.672		
V23 <--- F4	0.623	V40 <--- F8	0.890	V43 <--- F9	0.755
V24 <--- F4	0.541	V41 <--- F8	0.928	V44 <--- F9	0.784
V25 <--- F4	0.678	V42 <--- F8	0.923	V45 <--- F9	0.676
V26 <--- F4	0.582				
V36 <--- F7	0.646	V46 <--- F10	0.607	V49 <--- F11	0.615
V37 <--- F7	0.684	V47 <--- F10	0.631	V50 <--- F11	0.800
V38 <--- F7	0.714	V48 <--- F10	0.644	V51 <--- F11	0.620
V39 <--- F7	0.704			V52 <--- F11	0.635
V53 <--- F12	0.659	V56 <--- F13	0.681	V59 <--- F14	0.640
V54 <--- F12	0.764	V57 <--- F13	0.750	V60 <--- F14	0.710
V55 <--- F12	0.797	V58 <--- F13	0.740	V61 <--- F14	0.496
V62 <--- F15	0.479	V64 <--- F15	0.658	V66 <--- F15	0.769
V63 <--- F15	0.687	V65 <--- F15	0.592		

Corporate social responsibility model								
Factors	Variance Explained	Standard Percentage	Factors	Variance Explained	Standard Percentage	Factors	Variance Explained	Standard Percentage
F1	11.927	21.40	F6	3.045	5.46	F11	2.012	3.61
F2	9.508	17.06	F7	2.722	4.88	F12	1.933	3.47
F3	5.400	9.69	F8	2.461	4.42	F13	1.658	2.98
F4	4.014	7.20	F9	2.395	4.30	F14	1.636	2.94
F5	3.409	6.12	F10	2.050	3.68	F15	1.553	2.79
Total explained variance				55.723	Percent explained variance			100.00

**Fig 1:**
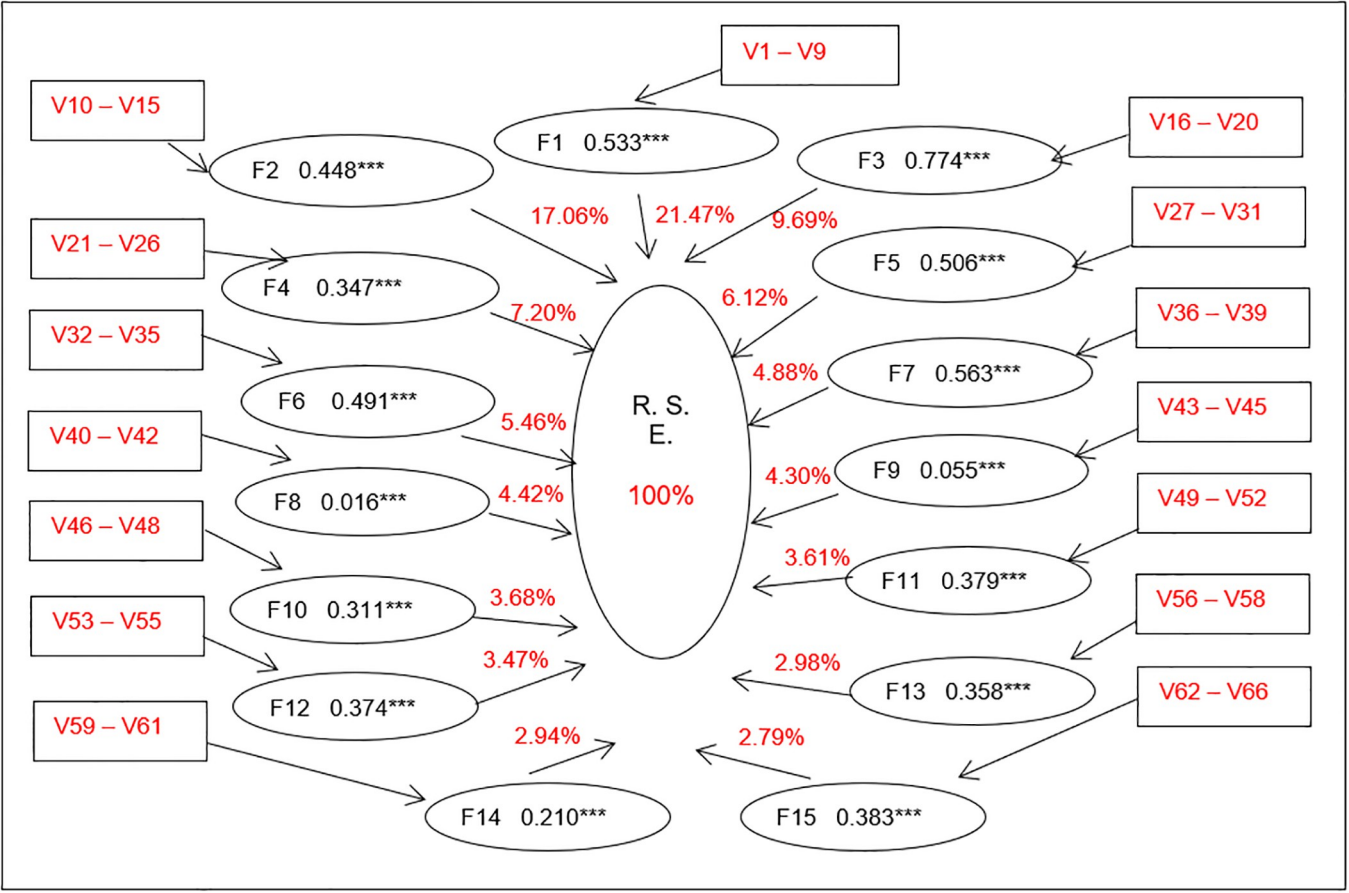
Factors contribution the CSR construct Ecuadorian sample of 667 cases.

Remarkably, the standardized variance indices of the variables and their respective factors show that 6 of the 15 factors contain at least three variables (F8, F9, F10, F12, F13, F14). These factors reach estimators that significantly ratify their respective conformations. In addition, the other factors contain between 9 items (F1) and 4 reagents (F6, F7, F11) and structure a statistically supported CSR model [32,33,36]. Furthermore, as shown in Fig 1, it determines of the percentage magnitude each factor contributes to the CSR model determined in the present study. For example, factor F1 integrates V1 to V9 items, followed by factor F2 with the variables V10 to V15 and so forth with the other factors that totalling 66 variables.

Synthesizing the analysis of the CSR model factors, each factor contribution in percentages ors was determined, as detailed in Table 4, from the factorial analysis results. Complementarily, the firgure 1 shows, in simplified form, the validation obtained through the covariance analysis.

Therefore, the first four factors of the AFE analysis explain 30.849% of the total variance, standardized at 100%, which is equivalent to 55.36% of the model’s total variance. The factor F1, Environment and ecology contributes 21.40%; F2, Job satisfaction provides 17.06%; F3, Business management, collaborates with 9.69% and F4, Production, does so with 7.2%, so these first four factors account for more than half of the social responsibility management concerns of business organizations. The other factors of the model contribute to varied percentages that range between 6.12% and 2.79%, totaling 100% of factors and variables that explain CSR in the Ecuadorian context of medium and large companies in Guayas, Ecuador.

### 3.5. Determination of covariance relationships of corporate social responsibility factors

Based on what was stated in the previous section, which confirms the relationships of the model variables with their respective factors [17], this section details the results of the analysis of covariance [31] that occur between the factors of the CSR that are studied here.

Consequently, it is necessary to mention that the analysis of covariance method itself [32] requires establishing, in its formulation, the relationships that occur between the 15 factors, since it is from the estimators that the method determines that it is possible to confirm reliably the factorial modeling that this work proposes. Finally, to verify how and to what extent the factors of the CSR model are related to give shape and content to a coherent set of CSR factors, Table 4 shows the results of the analysis of covariance among the 15 factors analyzed.

Fifteen CSR factors are mutually related with different degrees of significance (1% = ***, 5% = **, 10% = *). For example, the first four factors that establish the most significant number of relationships are F6, which covaries with 7 factors (F2, F4, F8, F9, F10, F13, and F14); F7 is also related with 7 factors (F2, F4, F5, F8, F10, F11, and F14); followed by F2 related to 5 factors (F8, F9, F10, F11, and F14); and factor F3 related to five factors (F1, F5, F6, F11, and F12). The other factors also establish mutual relationships in different magnitudes with between 1 (F12 and F13 both with F14) and 4 factors of the CSR model, as detailed in Table 4. Thus, for example, F1 is related to 4 factors, with F5, F11, and F12. It does so significantly at 1% (***) and with F13 at a slightly lower magnitude of 10% (**). In comparison, while factor F2 also does so with 5 factors, with factors F8, F9, F10 and F14 at a high magnitude of 1% (***) and F11 at 10% (**). Similarly, the other factors also present relevant covariance relationships, as detailed in Table 5.

**Table 5:** CSR factor covariance model Ecuadorian sample of 667 cases.

	Estimator	S.E.	Standard	P		Estimator	S.E.	Standard	P
F1 <--> F5	0.159	0.021	0.305	***	F2 <--> F8	0.184	0.027	0.273	***
F1 <--> F11	0.163	0.019	0.363	***	F2 <--> F9	0.120	0.029	0.174	***
F1 <--> F12	0.089	0.018	0.199	***	F2 <--> F10	0.103	0.019	0.276	***
F1 <--> F13	-0.031	0.018	-0.071	0.084*	F2 <--> F11	0.031	0.017	0.076	0.063*
					F2 <--> F14	0.051	0.015	0.165	***
F3 <--> F1	0.171	0.024	0.266	***	F4 <--> F2	0.097	0.016	0.247	***
F3 <--> F5	0.139	0.027	0.223	***	F4 <--> F8	0.065	0.023	0.110	0.004**
F3 <--> F6	-0.046	0.024	-0.075	0.059*	F4 <--> F9	0.263	0.031	0.435	***
F3 <--> F11	0.401	0.028	0.740	***	F4 <--> F10	0.078	0.016	0.239	***
F3 <--> F12	0.090	0.022	0.168	***	F4 <--> F14	0.052	0.013	0.191	***
F5 <--> F11	0.199	0.020	0.455	***	F6 <--> F2	0.033	0.020	0.070	0.095*
F5 <--> F12	0.106	0.019	0.244	***	F6 <--> F4	0.139	0.019	0.337	***
F7 <--> F2	0.232	0.025	0.463	***	F6 <--> F8	0.191	0.030	0.270	***
F7 <--> F4	0.076	0.020	0.172	***	F6 <--> F9	0.298	0.035	0.414	***
F7 <--> F5	0.091	0.024	0.170	***	F6 <--> F10	0.184	0.022	0.470	***
F7 <--> F8	0.289	0.036	0.382	***	F6 <--> F13	0.042	0.020	0.099	0.033**
F7 <--> F10	0.077	0.024	0.184	0.001**	F6 <--> F14	0.055	0.018	0.172	0.002**
F7 <--> F11	0.063	0.022	0.137	0.004**	F8 <--> F9	0.184	0.043	0.177	***
F7 <--> F14	0.101	0.019	0.295	***	F8 <--> F10	0.149	0.028	0.265	***
F9 <--> F10	0.175	0.032	0.305	***	F8 <--> F13	0.065	0.026	0.108	0.011**
F9 <--> F14	0.078	0.024	0.165	0.001**	F8 <--> F14	0.057	0.022	0.123	0.008**
F11 <--> F12	0.103	0.017	0.272	***	F10 <--> F13	0.043	0.018	0.130	0.018**
F11 <--> F14	-0.039	0.014	-0.137	0.006**	F10 <--> F14	0.067	0.015	0.261	***
F13 <--> F14	0.040	0.015	0.146	0.008**	F12 <--> F14	-0.024	0.014	-0.087	0.088*
F15 <--> F1	0.149	0.020	0.329	***	F15 <--> F11	0.221	0.019	0.580	***
F15 <--> F3	0.212	0.024	0.390	***	F15 <--> F12	0.248	0.020	0.655	***
F15 <--> F5	0.182	0.020	0.415	***	F15 <--> F14	-0.048	0.015	-0.170	***
Goodness of fit of analysis of covariances									
ÍndicesCMIN				NPAR	CMIN	DF	CMIN/DF	P	CFI
Default model				362	3670.918	1915	1.917	0.000	0.908
Saturated model				2277	0.000	0	-	-	1.000
Independence model				132	21210.947	2145	9.889	0.000	0.000
ÍndicesBaseline Comparisons				PRATIO	RMSEA	NFI Delta1	IFI Delta2	TLI rho2	
Default model				0.893	0.037	0.827	0.909	0.897	
Saturated model				0.000	0.116	1.000	1.000	-	
Independence model				1.000	-	0.000	0.000	0.000	

Nota: S.E. = Standard deviation; P = Stadistical significance (***).

Next, to verify the construct validity, the goodness-of-fit indices [31] were determined through the set of confirmatory estimators of the covariance modelling of the CSR factors.

The CMIN/DF coefficient synthesized the first goodness-of-fit estimator, which shows a significant value of 1.917 (***) below the maximum required estimator (≤ 3). This indicator is validated through the CFI comparative adjustment coefficient, which reaches a ratifying estimator of 0.908 above the expected value (≥ 0.90). Also, the estimate of the mean square error of approximation, RMSEA, presents a confirmatory index of 0.037, below the maximum acceptable (≤ 0.05). Hence, the index corroborated the adjustment of the 15 factors and 66 items of the updated CSR model proposed in this work.

### 3.6. Confirmatory analysis in a Chilean contrast sample

Accepting the methodological orientations that suggest confirming a factorial modeling in an alternative contrast sample [18,19], it was decided to verify the model of 15 factors [17], determined in the Ecuadorian reality, using a sample of 1100 cases obtained in the Chilean context in order to ratify the statistical behavior of the estimators previously obtained [31]. For this, the structural analysis was carried out with this new sample, applying the covariance method [34], intended to ratify the inclusion of the variables to their respective factors [17,18] and which allowed determining the estimators detailed in the Table 6.

**Table 6:** Confirmatory model of CSR in the alternative contrast sample Chilean sample of 1100 cases.

Variances	Estimators	Variances	Estimators	Variances	Estimators
V1<---F1	0.677	V10<---F2	0.641	V16<---F3	0.737
V2<---F1	0.696	V11<---F2	0.839	V17<---F3	0.653
V3<---F1	0.701	V12<---F2	0.649	V18<---F3	0.728
V4<---F1	0.649	V13<---F2	0.757	V19<---F3	0.846
V5<---F1	0.658	V14<---F2	0.748	V20<---F3	0.651
V6<---F1	0.637	V15<---F2	0.696		
V7<---F1	0.594	V27<---F5	0.707	V32<---F6	0.577
V8<---F1	0.617	V28<---F5	0.716	V33<---F6	0.646
V9<---F1	0.66	V29<---F5	0.774	V34<---F6	0.774
V21<---F4	0.653	V30<---F5	0.752	V35<---F6	0.595
V22<---F4	0.641	V31<---F5	0.683		
V23<---F4	0.62	V40<---F8	0.903	V43<---F9	0.748
V24<---F4	0.569	V41<---F8	0.919	V44<---F9	0.788
V25<---F4	0.676	V42<---F8	0.891	V45<---F9	0.692
V26<---F4	0.567	V46<---F10	0.586	V49<---F11	0.725
V36<---F7	0.669	V47<---F10	0.575	V50<---F11	0.614
V37<---F7	0.746	V48<---F10	0.609	V51<---F11	0.59
V38<---F7	0.779			V52<---F11	0.64
V39<---F7	0.739	V56<---F13	0.662	V59<---F14	0.714
V53<---F12	0.728	V57<---F13	0.772	V60<---F14	0.451
V54<---F12	0.703	V58<---F13	0.713	V61<---F14	0.567
V55<---F12	0.748				
V62<---F15	0.55	V64<---F15	0.658	V66<---F15	0.706
V63<---F15	0.703	V65<---F15	0.54		

Corporate social responsibility model									
Factor	Estimators	S.E.	C.R.	P	Factor	Estimators	S.E.	C.R.	P
F1	0.523	0.025	20.946	***	F9	1.046	0.05	19.87	***
F2	0.426	0.019	21.961	***	F10	0.281	0.02	12.77	***
F3	0.89	0.041	21.547	***	F11	0.378	0.02	17.87	***
F4	0.372	0.02	18.867	***	F12	0.315	0.02	16.63	***
F5	0.483	0.024	20.377	***	F13	0.351	0.02	17.75	***
F6	0.476	0.025	19.323	***	F14	0.169	0.01	15.5	***
F7	0.609	0.03	20.047	***	F15	0.387	0.02	18.29	***
F8	1.018	0.048	21.183	***					
Goodness of fit of analysis of covariances									
**CMIN**				NPAR		CMIN	DF	P	**CMIN/DF**
Default model				628		5374.828	1649	0	**3.259**
Saturated model				2277		0	0		
Independence model				132		39258.818	2145	0	18.302
**Baseline Comparisons**				NFI Delta1		RFI rho1	IFI Delta2	TLI rho2	CFI
Default model				0.863		0.822	0.901	0.869	0.9
Saturated model				1			1		1
Independence model				0		0	0	0	0
**RMSEA**				RMSEA		LO 90	HI 90	PCLOSE	
Default model				0.045		0.044	0.047	1	
Independence model				0.125		0.124	0.127	0	

Nota: S.E. = Standard deviation; C.R. = Critical ratios; P = Stadistical significance (***).

The results of the confirmatory analysis of the factors [31] carried out with the alternative sample show that the 15 factors obtain significant estimators (***), which means that these findings are not the product of chance, but rather demonstrate that each one of them the factors include their respective variables with estimators similar to those obtained with the Ecuadorian sample. In addition to what is indicated and detailing the results, it can be observed that the CMIN/DF estimator presents a significant value of 3.259 (***) in addition to a comparative adjustment index CFI, which reaches a value of 0.900, and an error estimator quadratic. approximation average, RMSEA, which reaches 0.045. Consequently, the alternative model carried out with the Chilean sample of 1,100 cases ratifies the conformation of the 15 factors and validates the inclusion of the 66 variables that make up an updated model of CSR, which this paper seeks to propose (Table 6).

## 4. Discussion

The factorial analysis determined an integrated system of factors from duly validated items, as well as the identification of some items that did not achieve the expected persistence to form part of the CSR model studied here. The finding is consistent with similar studies available in the literature [37] that mention variables such as corporate reputation, purchase intention, voluntarism, commitment, sustainability, and organizational performance, stating that it is necessary to continue with theoretical development. and practice of CSR from the academy and that this work is carried out through this proposal. In the Latin American context, the studies address issues related to applied social responsibility, for example, at the sectoral level of universities [38] where qualitative approaches have been made to evaluate university social responsibility, RSU, based on empirical data oriented to decision-making in teaching, research and extension, by the way, through joint work with the community, among other sectors.

Regarding the methodologies used, various qualitative studies have analyzed the reality, formulating decision proposals that must be adopted at specific moments and contexts [39], recognizing, in the Peruvian case, the role of interest groups, non-governmental organizations and governments, as well as the media given the impact of the COVID-19 pandemic, highlighting the importance of sustainability, social aid, actions with workers and customers, which end up activating a rather reactive approach to CSR action.. On the one hand, organizations seek to meet social and environmental needs such as those determined in this work, which are equivalent to the factors determined in other studies [40] that prioritize reputation and social innovation, which are perceived as diffuse terms and ambiguous, but they do achieve consensus regarding social, environmental and quality of life needs, such as those suggested by the present study of factors.

Regarding the methodologies used, various qualitative studies have analyzed the reality, formulating decision proposals that must be adopted at specific moments and contexts [39], recognizing, in the Peruvian case, the role of interest groups, non-governmental organizations and governments., as well as the media given the impact of the COVID-19 pandemic, highlighting the importance of sustainability, social aid, actions with workers and customers, which end up activating a rather reactive approach to CSR action.. On the one hand, organizations seek to meet social and environmental needs such as those determined in this work, which are equivalent to the factors determined in other studies [40] that prioritize reputation and social innovation, which are perceived as diffuse terms and ambiguous, but they do achieve consensus regarding social, environmental and quality of life needs, such as those suggested by the present study of factors.

In general, the factors F1 Environment and ecology and F10 Environment management of the present work allow us to identify foreseeable adverse effects to those discovered by Rengifo [41] who concludes in the need to achieve a deeper awareness in order to manage globalization and its effects. on the planet, forcing companies to act responsibly, which entails the need to analyze the link between CSR, with Science, Technology and its incidence on Society [42]. Complementarily, the components of the factors F2 Work satisfaction, F5 Working conditions and F9 People Management, highlight the role of boards, whose functions include promoting participation and diversity in order to promote governance [43], by the way, within the framework of the current regulations and with the territories in which the organizations operate [44] taking care of the global dimensions as well as the endogenous factors that fit the organizations.

From the operational perspective of organizations, factor 4, referring to production, focuses on logistics, since, for example, it allows organizations to internalize the demands of international markets that become mandatory for exporting companies. These companies must implement, as has already been studied, differentiation strategies applying CSR protocols at the process and product level [45], highlighting, as an example, quality attributes through organic certifications to promote internationalization and incursion into markets. demanding, as well as the creation of a brand image and a positive reputation and, above all, strengthened differentiation from its competitors. Hence, the international requirements are also projected towards the working conditions addressed in factor 5 related to Working conditions to the extent that this includes, on the one hand, the characteristics and practices of a social nature and, on the other, the need to promote the exercise of a diversity of compensation and incentives methods, identified in factor 6 called Compensation and incentives, through which organizations are obliged to maintain and promote precisely updated practices that enhance CSR [46] while identifying traits characteristics that define or determine an innovative organizational culture in the social sphere.

Various dimensions, in accordance with the imaginary of participation and development referred to in factor 7, it is necessary to have some key descriptors of social responsibility required by subordinate groups and internal managers of organizations [47] among which are mention the dimensions of ethical discernment, community, environment and imaginary of social responsibility, as is the case studied among companies in the health sector. Next, regarding the management of human talents, factor 8 highlights the importance of training activities and development of human skills that are adequately detailed by the CSR components, because it is through this factor that I manifest the importance of professional training, confirming the results of other works that positively and significantly correlate the development of people with social responsibility as it was studied in the context of postgraduate professionals in Peru [48]. Likewise, the present work contributes to the better management of people, as evidenced by factor 9 referred to People Management that describes what the necessary efforts must be, to strengthen human talent and enhance its development, confirming the findings of other investigations carried out in Colombia [49], where companies have sought to strengthen themselves internally by investing in their personnel, precisely in the perspective of globalization to successfully venture into the international arena, additionally, factor 12, labor benefits, refers to the fact that Organizations must guarantee a diversity of benefits for their workers and middle managers, given the wide international demand for this key component of CSR [50].

So too, the components of the tenth factor, called environmental management, and of factor 11, business management, progressively become one of the organizational ways to achieve some competitive advantage that can be successful and achieve the greatest impact on the corporate image of the company. in the short and long term. long-term in current and future markets [51]. Within these markets, factor 13 is identified, referring to the supply markets and internal purchasing processes, which must be adequately managed under the limitations presented by the components of factor 14 related to the economic situation. The financial aspect requires evaluating and reassessing the incidence of CSR on the sources and uses of available financing to improve the welfare of society, as has been demonstrated in the savings market [5]. Factor 15 includes external concerns of organizations, such as family benefits that make CSR effective through the well-being of the families of employees.

Finally, the structural modeling [31] carried out with the alternative sample obtained in the Chilean context, allows confirming the 15 CSR factors [17,19] determined in the Ecuadorian reality since the factors are made up of the 66 variables that are defined as persistent, given their factor load and communality estimators, and which are confirmed by structural analysis [36]. Additionally, from the goodness-of-fit estimators it can be seen that the CMIN/DF estimator [32] shows a significant value of 3.259 (***) in addition to a comparative fit index CFI, which reaches the expected value of 0.90 in addition to a root mean square error index of approximation, RMSEA, which reaches 0.045 [19]. Consequently, the alternative model carried out with the Chilean sample of 1100 cases ratifies the conformation of the 15 factors and validates the inclusion of the 66 variables that shape an updated model of CSR, which the present study proposes.

## 5. Conclusions

In the first place, the study was carried out using a rigorous and systematic methodology to identify the interrelationships between a large number of elements that included 104 variables and 17 factors using confirmatory factorial and structural modeling that allows proposing a new system of representative dimensions of social responsibility. business in the Ecuadorian context and that can be used as a guide for the management of organizations. Likewise, the work carried out contributes to the understanding of the management of organizations, provides a theoretical and empirical framework to identify, understand and measure some key variables and, by the way, contributes to business decision-making by specifying a system of dimensions such as a tool to guide social responsibility strategies and improve their performance in this area.

The set of variables of the factorial model was sensitized, verifying the communality and factor load of the 104 variables, confirming the validity of 66 variables and eliminating 38 items that failed to persist given the required statistical requirements, consequently, they did not meet the statistical requirements. necessary and demonstrate the rigor and consistency of the analysis. By discarding the variables that did not remain consistent, the quality and reliability of the resulting factorial model is improved.

H1 is validated since it was possible to determine an integrated system of 15 factors that identify key dimensions of CSR, forming a model of 66 variables that seek to guide the management of organizations and that explain 55.723% of the total variance. This implies that the factorial model developed is capable of capturing and explaining essential aspects of corporate social responsibility in the study context. In addition, the total explained variance provides a measure of the explanatory power of the factorial model. This percentage indicates that the model is capable of explaining a substantial part of the variability observed in the included variables, which reinforces its validity and relevance.

The factorial system of the CSR model and its respective variables, after being analyzed by means of the covariance method in the sample of 667 Ecuadorian cases and the 1100 cases of the alternative sample of the Chilean context, demonstrate the breadth of the sample and improve the generalization of the results. A large and representative sample size provides a solid basis for drawing conclusions about the existence and validity of the proposed CSR model. Additionally, since the analysis was carried out in two different realities (Ecuador and Chile) that confirm the existence of a coherent model of CSR factors, the H2 hypothesis is confirmed, since the identified factors are consistent and relevant both in the Ecuadorian context as in the Chilean This is supported by ratifying goodness-of-fit estimators such as CMIN/DF (quotient between the Chi-square statistic and the degrees of freedom), CFI (Comparative Fit Index) and RMSEA (Root Mean Square Error of Approximation)., whose indices positively evaluate the findings and confirm how well the model obtained fits the data. By mentioning that these estimators reach the required confirmation values, it is indicated that the CSR model adequately fits the data in both contexts and is statistically valid.

Finally, the factorial and structural modeling work carried out in two different realities facilitates the option of generalization and applicability of the results obtained in this study, considering the fact that they may have broader implications in the academic and practical field. Other researchers and practitioners can use the findings to conduct additional research or apply the dimension system in different geographic or industrial contexts. The study provides an academic contribution by developing a factorial model and a system of representative dimensions of CSR and have significant theoretical and practical implications in the management of organizations and business decision-making, as well as in the generation of knowledge and the promotion of the corporate social responsibility.

## Supporting information

S1 File (DOCX)

S1 Data (SAV)

## References

[1] Carroll AB. Corporate social responsibility (csr) and corporate social performance (csp). In Kolb R. W., The SAGE Encyclopedia of Business Ethics and Society (pp. 746–754). Thousand Oaks: SAGE Publications, Inc. 2018.

[2] Wulf, BE. Responsabilidad Social Empresarial, Editorial Universidad de la Serena, ISBN: 9789567052387 pp. 160. 2018.

[3] Johnson-Cramer ME. Stakeholder Theory. In Kolb R. W., The SAGE Encyclopedia of Business Ethics and Society. 3247–3255. Thousand Oaks: SAGE Publications, Inc. 2018.

[4] Bowie NE. Rawls’s Theory of Justice. In Kolb R. W, The SAGE Encyclopedia of Business Ethics and Society (pp. 2862–2865). Thousand Oaks: SAGE Publications, Inc. 2018.

[5] Vásquez MFR, Dueñas GAH. Levels of corporate social responsibility in the Manabita Teaching Savings and Credit Cooperative. Dominio de las Ciencias. 2022, 8(1), 871–886. Available from https://dialnet.unirioja.es/servlet/articulo?codigo=8383399.

[6] Sen A. The Idea of Justice. Cambridge, Massachusetts: Harvard University Press. 2009.

[7] Roemer JE, Trannoy A. Equality of Opportunity: Theory and Measurement. J. Econ. Liter. 2016, 54(4), 1288–1332. doi: 10.1257/jel.20151206

[8] Locke EA. What is job satisfaction? Organ. Behav. Hum. Perform. 1969, 4(4), 309–336. doi: 10.1016/0030-5073(69)90013-0

[9] Hobbes T. (1987). 1651.

[10] Bustamante Ubilla MA, Toscanini Segale M, Mera Ortiz W, Lapo Maza M. Foundations And Origin Of Corporate Social Responsibility. Guayaquil: Ed. Publications Department Catholic University of Santiago de Guayaquil. 2018.

[11] Narveson J. Social contract theory. In Kolb R. W., The SAGE Encyclopedia of Business Ethics and Society (pp. 3139–3146). Thousand Oaks: SAGE Publications, Inc. 2018.

[12] Bustamante-Ubilla M. Evolution of the Corporate Social Responsibility Model. Empresarial. 2018, 12(46), 32–41. doi: 10.23878/empr.v12i46.140

[13] Sullivan LE. Ethics (Communication). In Sullivan L. E, The SAGE glossary of the social and behavioral sciences (pp. 184–185). Thousand Oaks: SAGE Publications, Inc. 2009a.

[14] Phillips R. Stakeholder theory and organizational ethics. Berrett-Koehler Publishers. 2003.

[15] Salazar AL, Hidalgo JFO, Manríquez MR. The corporate social responsibility from the perception of human capital. A case study. Contabilidad-Spanish Acc Rev. 2017, 20(1), 36–46. doi: 10.1016/j.rcsar.2016.01.001

[16] Freiberg HA, Stover JB, de la Iglesia G, Fernández LM. Polychoric and Tetrachoric Correlations in Exploratory and Confirmatory Factorial Studies, Prensa Médica Latinoamericana, Ciencias Psicológicas. 2013, 7(2), 151–164.

[17] Lorenzo-Seva U, Ferrando PJ. FACTOR 9.2: A comprehensive program for fitting exploratory and semiconfirmatory factor analysis and IRT models. Appl Psych Measur. 2013, 37–6, 497–498. Available from. http://apm.sagepub.com.

[18] Izquierdo I, Olea J, Abad FJ. Exploratory factor analysis in validation studies: Uses and recommendations, Psicothema. 2014, 26(3), 395–400. doi: 10.7334/psicothema2013.349 25069561

[19] Beavers AS, Lounsbury JW, other 4 authors. Practical considerations for using exploratory factor analysis in educational research. Pract Assess Res Eval. 2013, 18(6). doi: 10.7275/qv2q-rk76

[20] Bustamante Ubilla, MA. Elaboración de un modelo de balance de responsabilidad empresarial para las empresas de la Séptima Región de Maule, Chile. 1996. Tesis Doctoral, Universidad de Deusto, San Sebastián, España.

[21] Tapia Bonifaz AG, Gavilánez Vega M, Jácome Tamayo S, Balseca Castro J. La Responsabilidad Social Empresarial: un desafío para la sostenibilidad de las empresas del Ecuador. *3c Empresa*: *Investigación y Pensamiento Crítico*. 2018, 68–89. Available from. https://dialnet.unirioja.es/servlet/articulo?codigo=6708534.

[22] Carroll AB. Corporate social responsibility (csr) and corporate social performance (csp). In Kolb R. W, The SAGE Encyclopedia of Business Ethics and Society (pp. 746–754). Thousand Oaks: SAGE Publications, Inc. 2018.

[23] Tiep LT, Huan NQ, Hong TTT. Effects of corporate social responsibility on SMEs’ performance in emerging market. Cogent Bus. Manag. 2021, 8(1), 1878978. doi: 10.1080/23311975.2021.1878978

[24] Hu Q, Zhu T, Lin CL, Chen T, Chin T. Corporate social responsibility and firm performance in china’s manufacturing: A global perspective of business models. Sustainability (Switzerland). 2021, 13(4), 1–17. doi: 10.3390/su13042388

[25] Bernardi R. A., & Threadgill V. H. (2011). Women Directors and Corporate Social Responsibility. *EJBO*: *Electronic Journal of Business Ethics and Organizational Studies*, 15–21. Available from http://urn.fi/URN:NBN:fi:jyu-201201301096.

[26] Zhang JQ, Zhu H, Ding HB. Board composition and corporate social responsibility: An empirical investigation in the post Sarbanes-Oxley era. J. Bus. Ethics. 2013, 114(3), 381–392. doi: 10.1007/s10551-012-1352-0

[27] Medina-Vicent M. Gender Social Responsibility and moral obligation. *FEMERIS*: Revista Multidisciplinar de Estudios de Género. 2017, 30–48. doi: 10.20318/femeris.2017.3546

[28] Timmerman M, Lorenzo-Seva U. Dimensionality Assessment of Ordered Polytomous Items with Parallel Analysis. Psychological Methods. 2011, 16(2), 209–220. doi: 10.1037/a0023353 21500916

[29] Zavaleta-de Arma M, Brito-Carrillo LE, Garzón-Castrillo MA. Methodology to estimate and evaluate a knowledge management model using structural equations. Orinoquia. 2020, 24(1), 94–110. doi: http%3A//doi.org/10.22579/20112629.595

[30] Forero DE, Gómez A. Comparison of measurement models based on expectations and perceived performance for the satisfaction study in health services. Suma Psicológica. 2017, 24(2), 87–96. doi: 10.1016/j.sumpsi.2017.06.002

[31] Marsh H. W., Morin A. J. S., Parker P. D. & Kaur G. (2014). Exploratory structural equation modeling: An integration of the best features of exploratory and confirmatory factor analysis. *Annual Review of Clinical Psychology*, 10,85–110. doi: 10.1146/annurev-clinpsy-032813-153700 24313568

[32] Bandalos DL, Finney, SJ. Factor Analysis: Exploratory and Confirmatory. En G. R. Hancock y R. O. Mueller (Eds.), Reviewer’s guide to quantitative methods. Routledge: New York. 2010.

[33] Costello AB, Osborne J. Best practices in exploratory factor analysis: four recommendations for getting the most from your analysis. Pract. Asses. Res. Eval. 2005, 10(7), 1–9. Available from: http://pareonline.net/gettvn.asp?v=10&n=7.

[34] Muthen B, Asparouhov T. Bayesian SEM: A more flexible representation of substantive theory. Psychological Methods. 2013 17 (3) 313–335. doi: 10.1037/a0026802 22962886

[35] Budsaratragoon P, Jitmaneeroj B. Measuring causal relations and identifying critical drivers for corporate sustainability: The quadruple bottom line approach. Math Biosci Eng. 2019, 23(3), 292–316. doi: 10.1108/MBE-10-2017-0080

[36] Hogarty KY, Hines CV, Kromrey JD, Ferron JM, Munford KR. The quality of factor solutions in exploratory factor analysis: The influence of sample size, communality, and overdetermination. Edu. Psych. Measur. 2005, 65, 202–226. doi: 10.1177/0013164404267287

[37] Jaimes-Valdez MA, Jacobo-Hernández CA, Ochoa-Jiménez S. The benefits of corporate social responsibility: a literary review, Tiempo & Economía. 2021, 8(2), 201–217. doi: 10.21789/24222704.1720

[38] León GDCO, Cappello OSA Rodríguez OAP Orta SYM, Zarazua REA. University Social Responsibility and its implementation: a panoramic review. Emerging Trends in Education. 2022, 4(8), 163–190. doi: 10.19136/etie.a4n8.4756

[39] Hernández J, Yagui V. Corporate social responsibility performance disclosed in virtual media due to the impact of COVID-19. Cuadernos Latinoamericanos de Administración, 2021, 17(33). doi: 10.18270/cuaderlam.v17i33.3389

[40] Vargas-Merino JA. Social innovation: new face of social responsibility? critical conceptualization from the university perspective. Revista de Ciencias Sociales, 2021, 27(2), 435–446. Available from. https://dialnet.unirioja.es/servlet/articulo?codigo=7927675.

[41] Rengifo Medina CN, Sánchez Segura SM, Obando Peralta EC. Corporate Social Responsibility and Sustainable Development: Reflections from Applied Ethics. Revista De Filosofía. 2022, 39(100), 409–420. doi: 10.5281/zenodo.5990284

[42] González Duarte JY, González Curbelo VB, Preciado Martínez M, Brito Hernández D, Abreus Mora JL. Corporate social responsibility: link Science, Technology and Society. Revista Científica, Cultura, Comunicación y Desarrollo. 2021, 6(3), 49–57. Available from https://rccd.ucf.edu.cu/index.php/aes/article/view/313.

[43] García Salazar Á, Echeverri Rubio A, Vieira Salazar JA. Corporate social responsibility and governance: A review. *Revista Universidad & Empresa*. 2021, 23(40), 1–26. doi: 10.12804/revistas.urosario.edu.co/empresa/a.9389

[44] Lorenzoni Escobar L. Corporate social responsibility in the jurisprudence of the Colombian Constitutional Court: dimensions of obligation in voluntariness. Revista de Estudios Socio-Jurídicos. 2021, 23(1), 347–369. doi: 10.12804/revistas.urosario.edu.co/sociojuridicos/a.9085

[45] Rodriguez Eugenio KR., Gallo Apolo JE, González Illescas ML, Carmenate Fuentes LP. Corporate social responsibility as a differentiation strategy for exporting companies. INNOVA Res J. 2021, 6(3), 171–189. doi: 10.33890/innova.v6.n3.2021.1832

[46] González Vega BG, Morales Vargas A, Marín Jimeno YI, Barajas Ruiz ML. Distance organizational culture practices from a corporate social responsibility perspective. Revista Innova ITFIP, 2021, 8 (1), 12–18. doi: 10.54198/innova08.02

[47] Severino-González P, Medina-Giacomozzi A, Muñoz-Huaracán S. Corporate social responsibility and health system: perception of health workers in Chile. Interciencia, 2021, 46(3), 126–132. Available from https://www.redalyc.org/journal/339/33966543006/33966543006.pdf.

[48] Torres ML, Alvarado LLP, Saldaña GGC, Alegría RV, Ramírez JV, Alarcón LV. Vocational training and its relationship with social responsibility. Ciencia Educativa y Estudios Instruccionales. (ISSN: 1556-5068, Social Science Research Network, ID SSRN: 4059939). 2022. 5(1). Perú. ID de artículo galileo_028-022-001-010.

[49] Canizales Muñoz L. D. (2021). Corporate social responsibility and strengthening human talent in organizations, RHS. Revista. Humanismo. Soc, 9(1), 5. Available from doi: 10.22209/rhs.v9n1a04

[50] Trillos KAB, León JAP. Literature review on human resources and corporate social responsibility. Revista de Ingenierías Interfaces, 2021, 4(1), 1–11. Available from https://revistas.unilibre.edu.co/index.php/interfaces/article/view/8244.

[51] Loja-Cedillo J, Vargas-Abad E, Sánchez-González I, Villavicencio-Rodas M. CSR as a competitive advantage: study on its influence on consumer behavior. *Digital Publisher CEIT*. 2022, 7(2), 306–325. doi: 10.33386/593dp.2022.2.1044

