# Supplementary material for: Model of dimensions and variables of corporate social responsibility updated through structural equations
Source: PLoS One. 2024 Jun 25;19(6):e0296761. doi: 10.1371/journal.pone.0296761 (PMC11198800; doi:10.1371/journal.pone.0296761)
Supplement: S1 File — (DOCX) [file pone.0296761.s001.docx]

**COMPLETE QUESTIONNAIRE**

**ORIGINAL CSR MODEL**

The ESPOL Polytechnic University of Ecuador is conducting a study on the dimensions of social responsibility in Guayas, Ecuador. Your authorization is requested to participate in this research project whose objective is Present an updated model of dimensions and related variables of corporate social responsibility, giving shape to a generic construct based on statistics. Your participation is completely voluntary, if you do not wish to participate, there will be no negative consequences. You can withdraw from the study at any time. The response is completely anonymous. There is no associated risk. If you have any questions, you can consult Wilmer Carvache-Franco whose email is: [wcarvach@espol.edu.ec](mailto:wcarvach@espol.edu.ec)

I agree to participate ❑

I don´t agree to participate ❑

**SECTION I: PERSONAL BACKGROUND**

1. **Sex (Mark with an X)**

Female

Male

1. **Age (Mark with an X)**

Less than 26 years 26 to 34 years 35 to 43 years

44 to 52 years 53 to 61 years Over 61 years

1. **Nivel Educacional (Mark with an X)**

Technique Professional Master

Doctorate (PhD) Specialization

1. **Marital Status (Mark with an X)**

Single Married Divorced Free Union Widowed

1. **Position (Mark with an X)**

Director/Manager Coordinator Assitant Other____________

1. **Years of Service (Mark with an X)**

Less than 4 years 4 to 10 years 11 to 16 years

17 to 22 years 23 to 30 years Over 30 years

1. **Type of contract (Mark with an X)**

Contract – Definitive appointment Occasional hiring

Professional services

1. **Institution**

Public Private

**SECTION II: IMPORTANCE OF THE VARIABLE GROUPS**

|  | **IMPORTANCE** | **LOW** | |  | **HIGH** | |
| --- | --- | --- | --- | --- | --- | --- |
| **VARIABLE GROUPS** | | **1** | **2** | **3** | **4** | **5** |
| **G1** | Financial Economic Situation |  |  |  |  |  |
| **G2** | Commercial situation |  |  |  |  |  |
| **G3** | Production |  |  |  |  |  |
| **G4** | Productivity |  |  |  |  |  |
| **G5** | Staff |  |  |  |  |  |
| **G6** | Customer service |  |  |  |  |  |
| **G7** | Local Community Relations |  |  |  |  |  |
| **G8** | Security |  |  |  |  |  |
| **G9** | Compensation and Incentives |  |  |  |  |  |
| **G10** | Work satisfaction |  |  |  |  |  |
| **G11** | Participation and Communications |  |  |  |  |  |
| **G12** | Training And Development |  |  |  |  |  |
| **G13** | Employment benefits |  |  |  |  |  |
| **G14** | Working conditions |  |  |  |  |  |
| **G15** | Business management |  |  |  |  |  |
| **G16** | Relations with the National and International Community |  |  |  |  |  |
| **G17** | Environment and ecology |  |  |  |  |  |

**SECTION III: IMPORTANCE OF VARIABLES**

| **IMPORTANCE** | | **LOW** | | |  | | **HIGH** | | |
| --- | --- | --- | --- | --- | --- | --- | --- | --- | --- |
| **GROUP 1** | **VARIABLE** | | **1** | **2** | | **3** | | **4** | **5** |
| **G1V1** | Economic structure | |  |  | |  | |  |  |
| **G1V2** | Rotation | |  |  | |  | |  |  |
| **G1V3** | Cost effectiveness | |  |  | |  | |  |  |
| **G1V4** | Performance | |  |  | |  | |  |  |
| **GROUP 2** | **VARIABLE** | | **1** | **2** | | **3** | | **4** | **5** |
| **G2V1** | Sales | |  |  | |  | |  |  |
| **G2V2** | Distribution | |  |  | |  | |  |  |
| **G2V3** | Around | |  |  | |  | |  |  |
| **G2V4** | Shopping | |  |  | |  | |  |  |
| **GROUP 3** | **VARIABLE** | | **1** | **2** | | **3** | | **4** | **5** |
| **G3V1** | Quality | |  |  | |  | |  |  |
| **G3V2** | Amount | |  |  | |  | |  |  |
| **G3V3** | stocks | |  |  | |  | |  |  |
| **G3V4** | costs | |  |  | |  | |  |  |
| **GROUP 4** | **VARIABLE** | | **1** | **2** | | **3** | | **4** | **5** |
| **G4V1** | Technical Productivity | |  |  | |  | |  |  |
| **G4V2** | Economic Productivity | |  |  | |  | |  |  |
| **G4V3** | Factor Productivity | |  |  | |  | |  |  |
| **GROUP 5** | **VARIABLE** | | **1** | **2** | | **3** | | **4** | **5** |
| **G5V1** | Template | |  |  | |  | |  |  |
| **G5V2** | Social climate | |  |  | |  | |  |  |
| **G5V3** | Training, Promotion and Security | |  |  | |  | |  |  |
| **G5V4** | Wages | |  |  | |  | |  |  |

|  | **IMPORTANCE** | | | **LOW** | | |  | | **HIGH** | |
| --- | --- | --- | --- | --- | --- | --- | --- | --- | --- | --- |
| **GROUP 6** | | **VARIABLE** | **1** | | **2** | **3** | | **4** | | **5** |
| **G6V1** | | Customer Judgment |  | |  |  | |  | |  |
| **G6V2** | | Product quality |  | |  |  | |  | |  |
| **G6V3** | | Relationship with customers |  | |  |  | |  | |  |
| **GROUP 7** | | **VARIABLE** | **1** | | **2** | **3** | | **4** | | **5** |
| **G7V1** | | Relationship With Public Administration |  | |  |  | |  | |  |
| **G7V2** | | Company image |  | |  |  | |  | |  |
| **G7V3** | | Participation in Community Activities |  | |  |  | |  | |  |
| **GROUP 8** | | **VARIABLE** | **1** | | **2** | **3** | | **4** | | **5** |
| **G8V1** | | Job Security |  | |  |  | |  | |  |
| **G8V2** | | Work Accident Prevention |  | |  |  | |  | |  |
| **G8V3** | | Occupational Diseases |  | |  |  | |  | |  |
| **G8V4** | | Temporary job |  | |  |  | |  | |  |
| **GROUP 9** | | **VARIABLE** | **1** | | **2** | **3** | | **4** | | **5** |
| **G9V1** | | Remuneration System |  | |  |  | |  | |  |
| **G9V2** | | Recognition of Merits |  | |  |  | |  | |  |
| **G9V3** | | System of Promotions and Promotions |  | |  |  | |  | |  |
| **G9V4** | | Advance System |  | |  |  | |  | |  |
| **G9V5** | | Staff Parties |  | |  |  | |  | |  |
| **G9V6** | | Cultural aspects |  | |  |  | |  | |  |
| **G9V7** | | Sport activities |  | |  |  | |  | |  |
| **G0V8** | | Punctuality In Payments |  | |  |  | |  | |  |
| **G0V9** | | Vacation |  | |  |  | |  | |  |
| **G9V10** | | Profit sharing |  | |  |  | |  | |  |
| **G9V11** | | Stock Option |  | |  |  | |  | |  |
| **G9V12** | | Partial Unemployment Compensation |  | |  |  | |  | |  |

|  | **IMPORTANCE** | | | **LOW** | | |  | | **HIGH** | |
| --- | --- | --- | --- | --- | --- | --- | --- | --- | --- | --- |
| **GRUPO** | | **VARIABLE** | **1** | | **2** | **3** | | **4** | | **5** |
| **G10V1** | | Work satisfaction |  | |  |  | |  | |  |
| **G10V2** | | Psychological Balance |  | |  |  | |  | |  |
| **G10V3** | | Work absenteeism |  | |  |  | |  | |  |
| **G10V4** | | Staff Turnover |  | |  |  | |  | |  |
| **GROUP** | | **VARIABLE** | **1** | | **2** | **3** | | **4** | | **5** |
| **G11V1** | | Labor Relations |  | |  |  | |  | |  |
| **G11V2** | | Right to Opinion |  | |  |  | |  | |  |
| **G11V3** | | Participation System |  | |  |  | |  | |  |
| **G11V4** | | Inside Information Magazine |  | |  |  | |  | |  |
| **G11V5** | | Communications |  | |  |  | |  | |  |
| **G11V6** | | Union life |  | |  |  | |  | |  |
| **G11V7** | | Treatment of Complaints |  | |  |  | |  | |  |
| **G11V8** | | Company Committees |  | |  |  | |  | |  |
| **GROUP** | | **VARIABLE** | **1** | | **2** | **3** | | **4** | | **5** |
| **G12V1** | | Training And Development |  | |  |  | |  | |  |
| **G12V2** | | Staff Development Expenses |  | |  |  | |  | |  |
| **G12V3** | | Homework Enrichment |  | |  |  | |  | |  |
| **G12V4** | | Holidays for Training |  | |  |  | |  | |  |
| **G12V5** | | Learning Programs |  | |  |  | |  | |  |
| **G12V6** | | Staff Progression Pathways |  | |  |  | |  | |  |
| **GROUP** | | **VARIABLE** | **1** | | **2** | **3** | | **4** | | **5** |
| **G14V1** | | Working conditions |  | |  |  | |  | |  |
| **G14V2** | | Shift Assignment |  | |  |  | |  | |  |
| **G14V3** | | Technology |  | |  |  | |  | |  |
| **G14V4** | | Duration Of The Day |  | |  |  | |  | |  |
| **G14V5** | | Resource Guarantee for Older Workers |  | |  |  | |  | |  |
| **G14V6** | | Women's Status |  | |  |  | |  | |  |

|  | **IMPORTANCE** | | | **LOW** | | |  | | **HIGH** | |
| --- | --- | --- | --- | --- | --- | --- | --- | --- | --- | --- |
| **GROUP** | | **VARIABLE** | **1** | | **2** | **3** | | **4** | | **5** |
| **G13V1** | | Health Fund |  | |  |  | |  | |  |
| **G13V2** | | Compensation Fund |  | |  |  | |  | |  |
| **G13V3** | | Special Bonus System |  | |  |  | |  | |  |
| **G13V4** | | Institutional Loans |  | |  |  | |  | |  |
| **G13V5** | | Housing Loan System |  | |  |  | |  | |  |
| **G13V6** | | Emergency Loans |  | |  |  | |  | |  |
| **G13V7** | | Birth, Marriage, e Allowances |  | |  |  | |  | |  |
| **G13V8** | | Permissions |  | |  |  | |  | |  |
| **G13V9** | | Retirements |  | |  |  | |  | |  |
| **G13V10** | | Staff Transportation |  | |  |  | |  | |  |
| **G13V11** | | Quotes |  | |  |  | |  | |  |
| **G13V12** | | Compensation Maternity leave |  | |  |  | |  | |  |
| **GROUP** | | **VARIABLE** | **1** | | **2** | **3** | | **4** | | **5** |
| **G15V1** | | Organization Quality |  | |  |  | |  | |  |
| **G15V2** | | Performance evaluation |  | |  |  | |  | |  |
| **G15V3** | | Corporate Responsibility Compliance |  | |  |  | |  | |  |
| **G15V4** | | Management Prestige |  | |  |  | |  | |  |
| **G15V5** | | Investigation and development |  | |  |  | |  | |  |
| **G15V6** | | Direct and Indirect Generated Employment |  | |  |  | |  | |  |
| **G15V7** | | Employment of Human Resources and Materials |  | |  |  | |  | |  |
| **G15V8** | | Company Profitability |  | |  |  | |  | |  |
| **G15V9** | | Company Expansion |  | |  |  | |  | |  |
| **G15V10** | | Responsibility Centers |  | |  |  | |  | |  |

|  | **IMPORTANCE** | | | **LOW** | | |  | | **HIGH** | |
| --- | --- | --- | --- | --- | --- | --- | --- | --- | --- | --- |
| **GROUP** | | **VARIABLE** | **1** | | **2** | **3** | | **4** | | **5** |
| **G16V1** | | Contribution to Government Standards |  | |  |  | |  | |  |
| **G16V2** | | Information Social Responsibility |  | |  |  | |  | |  |
| **G16V3** | | Company Prestige |  | |  |  | |  | |  |
| **G16V4** | | Social Responsibility of Advertising |  | |  |  | |  | |  |
| **G16V5** | | Paid taxes |  | |  |  | |  | |  |
| **G16V6** | | Financing of Social Works |  | |  |  | |  | |  |
| **G16V7** | | Community Wellness |  | |  |  | |  | |  |
| **G16V8** | | Consumer Satisfaction |  | |  |  | |  | |  |
| **G16V9** | | Foreign trade |  | |  |  | |  | |  |
| **G16V10** | | International Competitiveness |  | |  |  | |  | |  |
| **GROUP** | | **VARIABLE** | **1** | | **2** | **3** | | **4** | | **5** |
| **G17V1** | | Environmental Conservation |  | |  |  | |  | |  |
| **G17V2** | | Acidity or Alkalinity of the Waters |  | |  |  | |  | |  |
| **G17V3** | | Total Water Waste |  | |  |  | |  | |  |
| **G17V4** | | Total Waste and Disposal |  | |  |  | |  | |  |
| **G17V5** | | Pollution |  | |  |  | |  | |  |
| **G17V6** | | Threats to the Health of the Population |  | |  |  | |  | |  |
| **G17V7** | | Energy saving |  | |  |  | |  | |  |

**SECTION IV: IMPORTANCE OF INDICATORS**

|  | **IMPORTANCE** | | **LOW** | | |  | | **HIGH** | | |
| --- | --- | --- | --- | --- | --- | --- | --- | --- | --- | --- |
| **GROUP** | | **INDICATORS** | | **1** | **2** | | **3** | | **4** | **5** |
| **G1V1** | | Financial ndependence | |  |  | |  | |  |  |
|  |  | L/P Financial Balance | |  |  | |  | |  |  |
|  |  | C/P Financial Balance | |  |  | |  | |  |  |
|  |  | Degree of immobilization | |  |  | |  | |  |  |
|  |  | Semi liquidity | |  |  | |  | |  |  |
|  |  | Liquidity | |  |  | |  | |  |  |
|  |  | Credit Suppliers | |  |  | |  | |  |  |
| **G1V2** | | Investment | |  |  | |  | |  |  |
|  |  | Net worth | |  |  | |  | |  |  |
|  |  | Of Permanent Capitals | |  |  | |  | |  |  |
|  |  | Stock | |  |  | |  | |  |  |
| **G1V3** | | Investment | |  |  | |  | |  |  |
|  |  | Of Permanent Capitals | |  |  | |  | |  |  |
|  |  | Of own capital | |  |  | |  | |  |  |
|  |  | Of The Exploitation | |  |  | |  | |  |  |
| **G1V4** | | About Sales | |  |  | |  | |  |  |
|  |  | Stock Market Multiple | |  |  | |  | |  |  |
|  |  | Contribution margin | |  |  | |  | |  |  |
| **GROUP** | | **INDICATORS** | | **1** | **2** | | **3** | | **4** | **5** |
| **G2V1** | | Quota Achievement | |  |  | |  | |  |  |
|  |  | Sales Composition | |  |  | |  | |  |  |
|  |  | Marketing Ratio | |  |  | |  | |  |  |
|  |  | Cost of sale | |  |  | |  | |  |  |
|  |  | Effectiveness of a delegation | |  |  | |  | |  |  |
| **G2V2** | | Unit cost | |  |  | |  | |  |  |
|  |  | Commercial Loads Coefficient | |  |  | |  | |  |  |
|  |  | Distribution channels | |  |  | |  | |  |  |
| **G2V3** | | Market share | |  |  | |  | |  |  |
|  |  | Order-book | |  |  | |  | |  |  |
|  |  | Coverage Index | |  |  | |  | |  |  |
|  |  | Positioning | |  |  | |  | |  |  |
| **G2V4** | | Unit Cost of Purchases | |  |  | |  | |  |  |

|  | **IMPORTANCE** | | **LOW** | | |  | | **HIGH** | | |
| --- | --- | --- | --- | --- | --- | --- | --- | --- | --- | --- |
| **GROUP** | | **INDICATORS** | | **1** | **2** | | **3** | | **4** | **5** |
| **G3V1** | | Production Type | |  |  | |  | |  |  |
|  |  | Rejections | |  |  | |  | |  |  |
|  |  | Use of Raw Materials | |  |  | |  | |  |  |
| **G3V2** | | Production Level | |  |  | |  | |  |  |
|  |  | Product Level | |  |  | |  | |  |  |
|  |  | Product Innovation | |  |  | |  | |  |  |
|  |  | Production Efficiency | |  |  | |  | |  |  |
| **G3V3** | | Work in progress level | |  |  | |  | |  |  |
|  |  | Raw Material Consumption | |  |  | |  | |  |  |
|  |  | Stock Rotation | |  |  | |  | |  |  |
|  |  | Stock Volume | |  |  | |  | |  |  |
| **G3V4** | | Total Unit Cost | |  |  | |  | |  |  |
|  |  | Average cost | |  |  | |  | |  |  |
| **GROUP** | | **INDICATORS** | | **1** | **2** | | **3** | | **4** | **5** |
| **G4V1** | | Labor Performance | |  |  | |  | |  |  |
|  |  | Employment Effectiveness | |  |  | |  | |  |  |
|  |  | Immobilization Yield | |  |  | |  | |  |  |
|  |  | Energy efficiency | |  |  | |  | |  |  |
|  |  | Staff Employment | |  |  | |  | |  |  |
|  |  | Employment of the means of production | |  |  | |  | |  |  |
|  |  | Factor Performance | |  |  | |  | |  |  |
| **G4V2** | | Price Performance | |  |  | |  | |  |  |
|  |  | Variable Cost Performance | |  |  | |  | |  |  |
|  |  | Production Cost Yields | |  |  | |  | |  |  |
|  |  | Total Cost Performance | |  |  | |  | |  |  |
|  |  | Marginal Return | |  |  | |  | |  |  |

|  | **IMPORTANCE** | | **LOW** | | |  | | **HIGH** | | |
| --- | --- | --- | --- | --- | --- | --- | --- | --- | --- | --- |
| **GROUP** | | **INDICATORS** | | **1** | **2** | | **3** | | **4** | **5** |
| **G5V1** | | Staff Level by Centers | |  |  | |  | |  |  |
|  |  | Template Structure By Centers | |  |  | |  | |  |  |
|  |  | Squad Structure By Ages | |  |  | |  | |  |  |
|  |  | Template Structure By Groups | |  |  | |  | |  |  |
|  |  | Abnormality Index | |  |  | |  | |  |  |
| **G5V2** | | External Rotation | |  |  | |  | |  |  |
|  |  | Suggestions | |  |  | |  | |  |  |
|  |  | Complaints | |  |  | |  | |  |  |
|  |  | Potential Internal Rotation | |  |  | |  | |  |  |
|  |  | True Internal Rotation | |  |  | |  | |  |  |
|  |  | Absenteeism Hours | |  |  | |  | |  |  |
| **G5V3** | | Level of education | |  |  | |  | |  |  |
|  |  | Security level | |  |  | |  | |  |  |
|  |  | Rhythm of production | |  |  | |  | |  |  |
| **G5V4** | | Salary Adequacy | |  |  | |  | |  |  |
|  |  | Incentives | |  |  | |  | |  |  |
| **GROUP** | | **INDICATORS** | | **1** | **2** | | **3** | | **4** | **5** |
| **G6V1** | | Deterioration Complaints | |  |  | |  | |  |  |
|  |  | Complaints About Delivery Times | |  |  | |  | |  |  |
|  |  | After Sales Service Complaints | |  |  | |  | |  |  |
|  |  | Customer Response | |  |  | |  | |  |  |
| **G6V2** | | Returns (Number and Value) | |  |  | |  | |  |  |
|  |  | Factorial análisis | |  |  | |  | |  |  |
|  |  | Service level | |  |  | |  | |  |  |
|  |  | Service Efficiency | |  |  | |  | |  |  |
| **G6V3** | | Customer Development | |  |  | |  | |  |  |
|  |  | Customer Credit | |  |  | |  | |  |  |
|  |  | Commercial discounts | |  |  | |  | |  |  |

|  | **IMPORTANCE** | | **LOW** | | |  | | **HIGH** | | |
| --- | --- | --- | --- | --- | --- | --- | --- | --- | --- | --- |
| **GROUP** | | **INDICATORS** | | **1** | **2** | | **3** | | **4** | **5** |
| **G7V1** | | Public Administration Line of Credit | |  |  | |  | |  |  |
|  |  | Tax Compliance | |  |  | |  | |  |  |
|  |  | Degree of Collaboration | |  |  | |  | |  |  |
| **G7V2** | | Public relations | |  |  | |  | |  |  |
|  |  | Information by Media Type | |  |  | |  | |  |  |
|  |  | Reputation | |  |  | |  | |  |  |
|  |  | Financial Coverage | |  |  | |  | |  |  |
| **G7V3** | | External Activity of the Company | |  |  | |  | |  |  |
|  |  | Internal Activity of the Company | |  |  | |  | |  |  |
| **GROUP** | | **INDICATORS** | | **1** | **2** | | **3** | | **4** | **5** |
| **G8V1** | | Percentage | |  |  | |  | |  |  |
|  |  | Layoff rate | |  |  | |  | |  |  |
| **G8V2** | | Accident rate | |  |  | |  | |  |  |
|  |  | Severity rate | |  |  | |  | |  |  |
| **G8V3** | | Disease index | |  |  | |  | |  |  |
|  |  | Disease prevention funds | |  |  | |  | |  |  |
| **G8V4** | | Number of persons employed | |  |  | |  | |  |  |
|  |  | Temporary employment index | |  |  | |  | |  |  |

|  | **IMPORTANCE** | | **LOW** | | |  | | **HIGH** | | |
| --- | --- | --- | --- | --- | --- | --- | --- | --- | --- | --- |
| **GROUP** | | **INDICATORS** | | **1** | **2** | | **3** | | **4** | **5** |
| **G9V1** | | Number of times per month | |  |  | |  | |  |  |
|  |  | Resettable index | |  |  | |  | |  |  |
| **G9V2** | | Opportunity - Quality | |  |  | |  | |  |  |
|  |  | Opinion index | |  |  | |  | |  |  |
| **G9V3** | | Promotions percentage | |  |  | |  | |  |  |
|  |  | Opinion index | |  |  | |  | |  |  |
| **G9V4** | | Opinion index | |  |  | |  | |  |  |
|  |  | Funds used | |  |  | |  | |  |  |
| **G9V5** | | Percentage of people | |  |  | |  | |  |  |
|  |  | Opinion index | |  |  | |  | |  |  |
| **G9V6** | | Percentage of people | |  |  | |  | |  |  |
|  |  | Opinion index | |  |  | |  | |  |  |
| **G9V7** | | Percentage of people | |  |  | |  | |  |  |
|  |  | Opinion index | |  |  | |  | |  |  |
| **G0V8** | | Punctuality index | |  |  | |  | |  |  |
|  |  | Frequency percentage | |  |  | |  | |  |  |
| **G0V9** | | Opinion index | |  |  | |  | |  |  |
|  |  | Funds used | |  |  | |  | |  |  |
| **G9V10** | | Number of people | |  |  | |  | |  |  |
|  |  | Opinion index | |  |  | |  | |  |  |
| **G9V11** | | Number of people benefited | |  |  | |  | |  |  |
|  |  | Funds used | |  |  | |  | |  |  |
| **G9V12** | | Number of people benefited | |  |  | |  | |  |  |
|  |  | Funds used | |  |  | |  | |  |  |

|  | **IMPORTANCE** | | **LOW** | | |  | | **HIGH** | | |
| --- | --- | --- | --- | --- | --- | --- | --- | --- | --- | --- |
| **GROUP** | | **INDICATORS** | | **1** | **2** | | **3** | | **4** | **5** |
| **G10V1** | | Opinion index | |  |  | |  | |  |  |
|  |  | Funds used | |  |  | |  | |  |  |
| **G10V2** | | Opinion index | |  |  | |  | |  |  |
|  |  | Percentage of cases attended | |  |  | |  | |  |  |
| **G10V3** | | Absenteeism rate | |  |  | |  | |  |  |
|  |  | Lost days value | |  |  | |  | |  |  |
| **G10V4** | | Opinion index | |  |  | |  | |  |  |
|  |  | Turnover index | |  |  | |  | |  |  |
| **GROUP** | | **INDICATORS** | | **1** | **2** | | **3** | | **4** | **5** |
| **G11V1** | | Opinion index | |  |  | |  | |  |  |
|  |  | Number of conflicts | |  |  | |  | |  |  |
| **G11V2** | | Opinion index | |  |  | |  | |  |  |
|  |  | Number of committees | |  |  | |  | |  |  |
| **G11V3** | | Opinion index | |  |  | |  | |  |  |
|  |  | Number of committees | |  |  | |  | |  |  |
| **G11V4** | | Opinion index | |  |  | |  | |  |  |
|  |  | Magazine frequency | |  |  | |  | |  |  |
| **G11V5** | | Frequency percentage | |  |  | |  | |  |  |
|  |  | Opinion index | |  |  | |  | |  |  |
| **G11V6** | | Number of people | |  |  | |  | |  |  |
|  |  | Opinion index | |  |  | |  | |  |  |
| **G11V7** | | Complaint rate | |  |  | |  | |  |  |
|  |  | Opinion index | |  |  | |  | |  |  |
| **G11V8** | | Number of participating people | |  |  | |  | |  |  |
|  |  | Opinion index | |  |  | |  | |  |  |

|  | **IMPORTANCE** | | **LOW** | | |  | | **HIGH** | | |
| --- | --- | --- | --- | --- | --- | --- | --- | --- | --- | --- |
| **GROUP** | | **INDICATORS** | | **1** | **2** | | **3** | | **4** | **5** |
| **G12V1** | | Opinion index | |  |  | |  | |  |  |
|  |  | Staff percentage | |  |  | |  | |  |  |
| **G12V2** | | Opinion index | |  |  | |  | |  |  |
|  |  | Earmarked funds | |  |  | |  | |  |  |
| **G12V3** | | Innovative tasks index | |  |  | |  | |  |  |
|  |  | Opinion index | |  |  | |  | |  |  |
| **G12V4** | | Number of people benefited | |  |  | |  | |  |  |
|  |  | Funds Used | |  |  | |  | |  |  |
| **G12V5** | | Number of people benefited | |  |  | |  | |  |  |
|  |  | Funds used | |  |  | |  | |  |  |
| **G12V6** | | Number of people promoted | |  |  | |  | |  |  |
|  |  | Opinion index | |  |  | |  | |  |  |
| **GROUP** | | **INDICATORS** | | **1** | **2** | | **3** | | **4** | **5** |
| **G13V1** | | Allocated Funds | |  |  | |  | |  |  |
|  |  | Opinion index | |  |  | |  | |  |  |
| **G13V2** | | Opinion index | |  |  | |  | |  |  |
|  |  | Funds used | |  |  | |  | |  |  |
| **G13V3** | | Funds used | |  |  | |  | |  |  |
|  |  | Opinion index | |  |  | |  | |  |  |
| **G13V4** | | Opinion index | |  |  | |  | |  |  |
|  |  | Allocated funds | |  |  | |  | |  |  |
| **G13V5** | | Number of people benefited | |  |  | |  | |  |  |
|  |  | Funds used | |  |  | |  | |  |  |
| **G13V6** | | Percentage of people | |  |  | |  | |  |  |
|  |  | Opinion index | |  |  | |  | |  |  |
| **G13V7** | | Number of people | |  |  | |  | |  |  |
|  |  | Opinion index | |  |  | |  | |  |  |
| **G13V8** | | Percentage of people | |  |  | |  | |  |  |
|  |  | Days granted | |  |  | |  | |  |  |
| **G13V9** | | Funds used | |  |  | |  | |  |  |
|  |  | Number of people | |  |  | |  | |  |  |
| **G13V10** | | Number of people benefited | |  |  | |  | |  |  |
|  |  | Opinion index | |  |  | |  | |  |  |
| **G13V11** | | Stock funds | |  |  | |  | |  |  |
|  |  | Opinion index | |  |  | |  | |  |  |
| **G13V12** | | Number of people benefited | |  |  | |  | |  |  |
|  |  | Funds used | |  |  | |  | |  |  |

| **IMPORTANCE** | | **LOW** | |  | | **HIGH** | | |  |  |
| --- | --- | --- | --- | --- | --- | --- | --- | --- | --- | --- |
| **GROUP** | **INDICATORS** | | **1** | | **2** | | **3** | **4** | | **5** |
| **G14V1** | Investments | |  | |  | |  |  | |  |
|  | Opinion index | |  | |  | |  |  | |  |
| **G14V2** | Percentage of people | |  | |  | |  |  | |  |
|  | Opinion index | |  | |  | |  |  | |  |
| **G14V3** | Funds used | |  | |  | |  |  | |  |
|  | New technologies developed | |  | |  | |  |  | |  |
| **G14V4** | Average duration of the day | |  | |  | |  |  | |  |
|  | Opinion index | |  | |  | |  |  | |  |
| **G14V5** | Number of people benefited | |  | |  | |  |  | |  |
|  | Opinion index | |  | |  | |  |  | |  |
| **G14V6** | Opinion index | |  | |  | |  |  | |  |
|  | Women employed index | |  | |  | |  |  | |  |

|  | **IMPORTANCE** | | **LOW** | | |  | | **HIGH** | | |
| --- | --- | --- | --- | --- | --- | --- | --- | --- | --- | --- |
| **GROUP** | | **INDICATORS** | | **1** | **2** | | **3** | | **4** | **5** |
| **G15V1** | | Number of levels | |  |  | |  | |  |  |
|  |  | Opinion index | |  |  | |  | |  |  |
| **G15V2** | | Effectiveness percentage | |  |  | |  | |  |  |
|  |  | Opinion Index | |  |  | |  | |  |  |
| **G15V3** | | Funds used | |  |  | |  | |  |  |
|  |  | External opinion index | |  |  | |  | |  |  |
| **G15V4** | | External opinion index | |  |  | |  | |  |  |
|  |  | Internal opinion index | |  |  | |  | |  |  |
| **G15V5** | | Research and development funds | |  |  | |  | |  |  |
|  |  | Number of innovations | |  |  | |  | |  |  |
| **G15V6** | | Number of jobs created | |  |  | |  | |  |  |
|  |  | Opinion index | |  |  | |  | |  |  |
| **G15V7** | | Number of persons employed | |  |  | |  | |  |  |
|  |  | Total costs | |  |  | |  | |  |  |
| **G15V8** | | Evolution of the profitability index | |  |  | |  | |  |  |
|  |  | Distribution of dividends | |  |  | |  | |  |  |
| **G15V9** | | Investment index | |  |  | |  | |  |  |
|  |  | Number of branches | |  |  | |  | |  |  |
| **G15V10** | | Decentralization index | |  |  | |  | |  |  |
|  |  | Opinion index | |  |  | |  | |  |  |

|  | **IMPORTANCE** | | **LOW** | | |  | | **HIGH** | | |
| --- | --- | --- | --- | --- | --- | --- | --- | --- | --- | --- |
| **GROUP** | | **INDICATORS** | | **1** | **2** | | **3** | | **4** | **5** |
| **G16V1** | | External opinion index | |  |  | |  | |  |  |
|  |  | Funds used | |  |  | |  | |  |  |
| **G16V2** | | External opinion index | |  |  | |  | |  |  |
|  |  | Investments | |  |  | |  | |  |  |
| **G16V3** | | External opinion index | |  |  | |  | |  |  |
|  |  | Internal opinion index | |  |  | |  | |  |  |
| **G16V4** | | External opinion index | |  |  | |  | |  |  |
|  |  | Internal opinion index | |  |  | |  | |  |  |
| **G16V5** | | Opinion index | |  |  | |  | |  |  |
|  |  | Funds used | |  |  | |  | |  |  |
| **G16V6** | | External opinion index | |  |  | |  | |  |  |
|  |  | Funds used | |  |  | |  | |  |  |
| **G16V7** | | Charitable funds | |  |  | |  | |  |  |
|  |  | Opinion index | |  |  | |  | |  |  |
| **G16V8** | | Consumer permanence | |  |  | |  | |  |  |
|  |  | Opinion index | |  |  | |  | |  |  |
| **G16V9** | | Doleras generation | |  |  | |  | |  |  |
|  |  | Opinion index | |  |  | |  | |  |  |
| **G16V10** | | Dollar generation | |  |  | |  | |  |  |
|  |  | Opinion index | |  |  | |  | |  |  |
| **GROUP** | | **INDICATORS** | | **1** | **2** | | **3** | | **4** | **5** |
| **G17V1** | | Opinion index | |  |  | |  | |  |  |
|  |  | Prevention funds | |  |  | |  | |  |  |
| **G17V2** | | Acidity index | |  |  | |  | |  |  |
|  |  | Funds used | |  |  | |  | |  |  |
| **G17V3** | | Waste rate | |  |  | |  | |  |  |
|  |  | Opinion index | |  |  | |  | |  |  |
| **G17V4** | | Opinion index | |  |  | |  | |  |  |
|  |  | Calcination index | |  |  | |  | |  |  |
| **G17V5** | | Funds used | |  |  | |  | |  |  |
|  |  | External opinion index | |  |  | |  | |  |  |
| **G17V6** | | Number of people affected | |  |  | |  | |  |  |
|  |  | Prevention funds | |  |  | |  | |  |  |
| **G17V7** | | Energy saving | |  |  | |  | |  |  |
|  |  | Energy innovations | |  |  | |  | |  |  |
